# Identifying the causal effects of long-term exposure to PM_2.5_ and ground surface ozone on individual medical costs in China—evidence from a representative longitudinal nationwide cohort

**DOI:** 10.1186/s12916-023-02839-1

**Published:** 2023-04-03

**Authors:** Ke Ju, Liyong Lu, Jingguo Yang, Ting Chen, Tianjiao Lan, Zhongxin Duan, Zongyou Xu, En Zhang, Wen Wang, Jay Pan

**Affiliations:** 1 School of Public Health and Preventive Medicine, Monash University, Level 2, 553 St Kilda Road, Melbourne, VIC 3004 Australia; 2 Center for Health Management and Policy Research, School of Public Health, Cheeloo College of Medicine, Shandong University, Jinan, 250012 China; 3 HEOA Group, West China School of Public Health and West China Fourth Hospital, Sichuan University, Chengdu, 610041 China; 4 Department of Neurosurgery, West China Hospital, Sichuan University, Chengdu, 610041 China; 5 Institute for Healthy Cities and West China Research Center for Rural Health Development, Sichuan University, Chengdu, 610041 China; 6 Medical School, Hubei Minzu University, Enshi, 445000 China; 7 School of Government, Peking University, Beijing, 100871 China; 8 School of Public Administration, Sichuan University, Chengdu, 610041 China

**Keywords:** Air pollution, Medical costs, Causal models, Tobit model, China

## Abstract

**Background:**

There is little evidence on whether PM_2.5_ and ground surface ozone have consistent effects on increased individual medical costs, and there is a lack of evidence on causality in developing countries.

**Methods:**

This study utilized balanced panel data from 2014, 2016, and 2018 waves of the Chinese Family Panel Study. The Tobit model was developed within a counterfactual causal inference framework, combined with a correlated random effects and control function approach (Tobit-CRE-CF), to explore the causal relationship between long-term exposure to air pollution and medical costs. We also explored whether different air pollutants exhibit comparable effects.

**Results:**

This study encompassed 8928 participants and assessed various benchmark models, highlighting the potential biases from failing to account for air pollution endogeneity or overlooking respondents without medical costs. Using the Tobit-CRE-CF model, significant effects of air pollutants on increased individual medical costs were identified. Specifically, margin effects for PM_2.5_ and ground-level ozone signifying that a unit increase in PM_2.5_ and ground-level ozone results in increased total medical costs of 199.144 and 75.145 RMB for individuals who incurred fees in the previous year, respectively.

**Conclusions:**

The results imply that long-term exposure to air pollutants contributes to increased medical costs for individuals, offering valuable insights for policymakers aiming to mitigate air pollution’s consequences.

**Supplementary Information:**

The online version contains supplementary material available at 10.1186/s12916-023-02839-1.

## Background

Air pollution has long been recognized to have a wide range of effects on human health, and long-term exposure to air pollutants is associated with a range of adverse health outcomes, such as an increased risk of dementia [1], type 2 diabetes [2], and lung cancer [3], among others. These associations can lead to increased medical costs for individuals. One study in South Africa found that failure to meet the U.S. National Ambient Air Quality Standard caused $14 billion in premature death-related losses in 2012, equivalent to 2.2 percent of that year’s gross domestic product (GDP) [4].

China has adopted a number of measures to control air pollution in recent years and has made some progress; however, more improvements are expected in the future [5]. Not only did China still expose approximately 42% of its population to an annual average PM_2.5_ concentrations above 35 μg/m^3^ in 2018 [5], but ground surface ozone pollution is causing increasing concern [6, 7]. Moreover, it is estimated that approximately 1.24 million Chinese people lost their lives to air pollution in 2017 [8]. A previous study using provincial-level data from China estimated that PM_2.5_ could result in $25.2 billion in health expenditure in 2030, approximately 2% of GDP, in the absence of a PM_2.5_ pollution control policy [9].

However, studies exploring the causal relationship between long-term air pollution and individual medical costs based on large cohorts are lacking, especially in developing countries such as China. Further, a significant problem faced by the current literature is that the effects of air pollution on health outcomes are often endogenous [10, 11], and it is challenging to control for this endogeneity to verify a causal relationship [12, 13]. Another problem is that most of the existing studies have been conducted at the city level and lack individual-level evidence; this is particularly important as a significant proportion of the population does not spend any medical costs in real life due to their good health. Thus, failure to properly address this special distribution will lead to biased results [10]. Furthermore, people may respond differently to different types of air pollutants, such that some will be better protected than others [14, 15]. Accordingly, it is of interest to examine whether different patterns of pollutants have consistent causal effects on individual medical costs.

To this end, this study utilized balanced panel data from 2014, 2016, and 2018 waves of the Chinese Family Panel Study (CFPS), a large representative long-term nationwide cohort, to develop a Tobit regression model combined with correlated random effects and the control function method to explore the causal effect of long-term exposure to different types of air pollutants on individual medical costs. The findings of this study can inform policy recommendations for improved control of the medical costs associated with air pollution.

## Methods

### Study population

The participants of this study were drawn from the CFPS, a nationwide, representative longitudinal cohort of Chinese adults. Participants for the CFPS were sampled from 25 provinces/autonomous regions/municipalities and were selected using a stratified multistage probability strategy. The survey has been conducted every 2 years since 2010. A 5-year balanced panel of adults (*N* = 8928) aged 18 years or older, who had never moved out of their district/county of residence, and who were interviewed in 2014, 2016, and 2018 were selected for this study. The county/district coverage of the sample and the number of participants within each county/district were shown in Additional file 1: Fig. S1-S3.

**Fig. 1:**
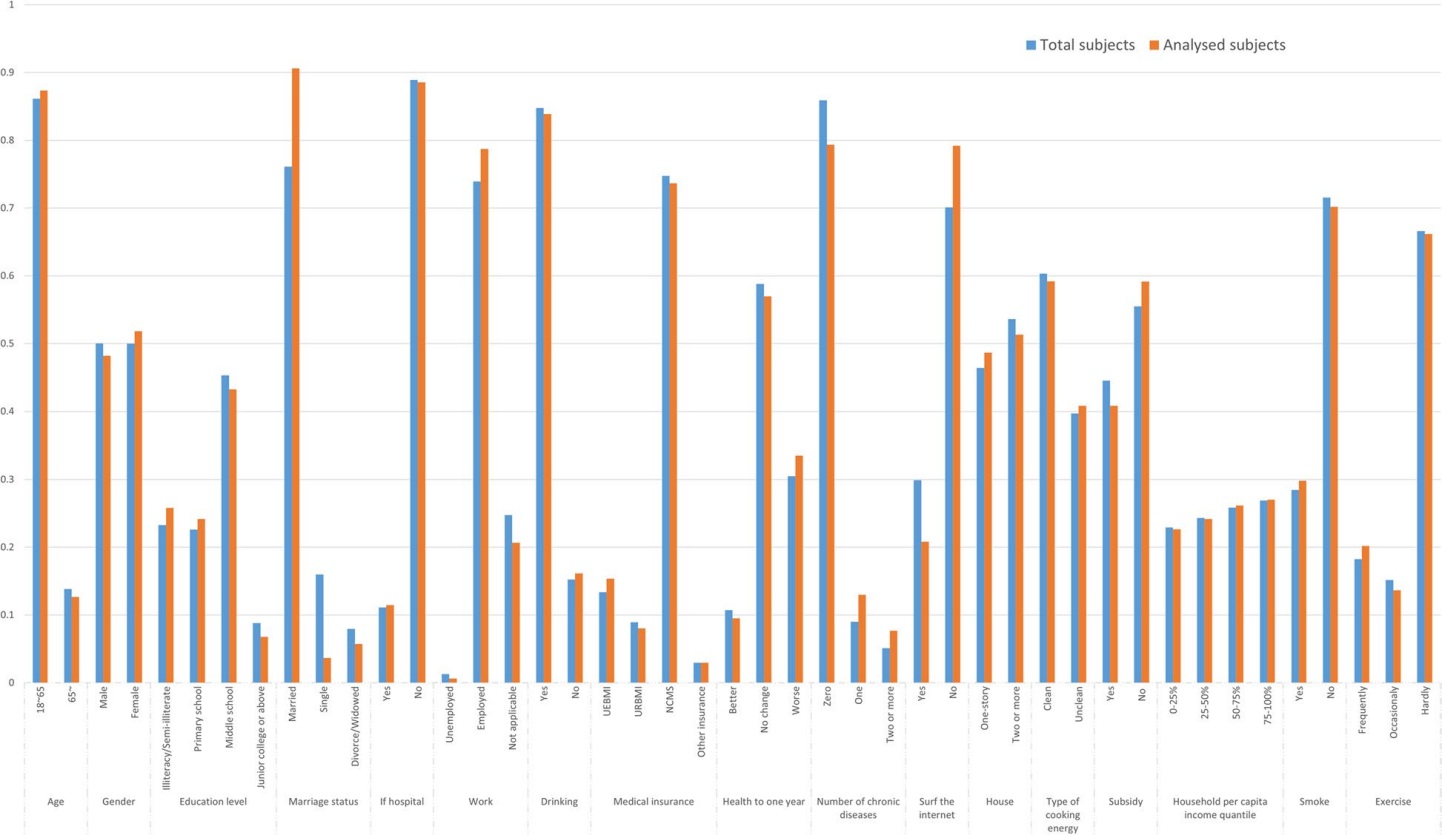
Characteristics of participants in this study compared to the full surveyed population in CFPS 2014

**Fig. 2:**
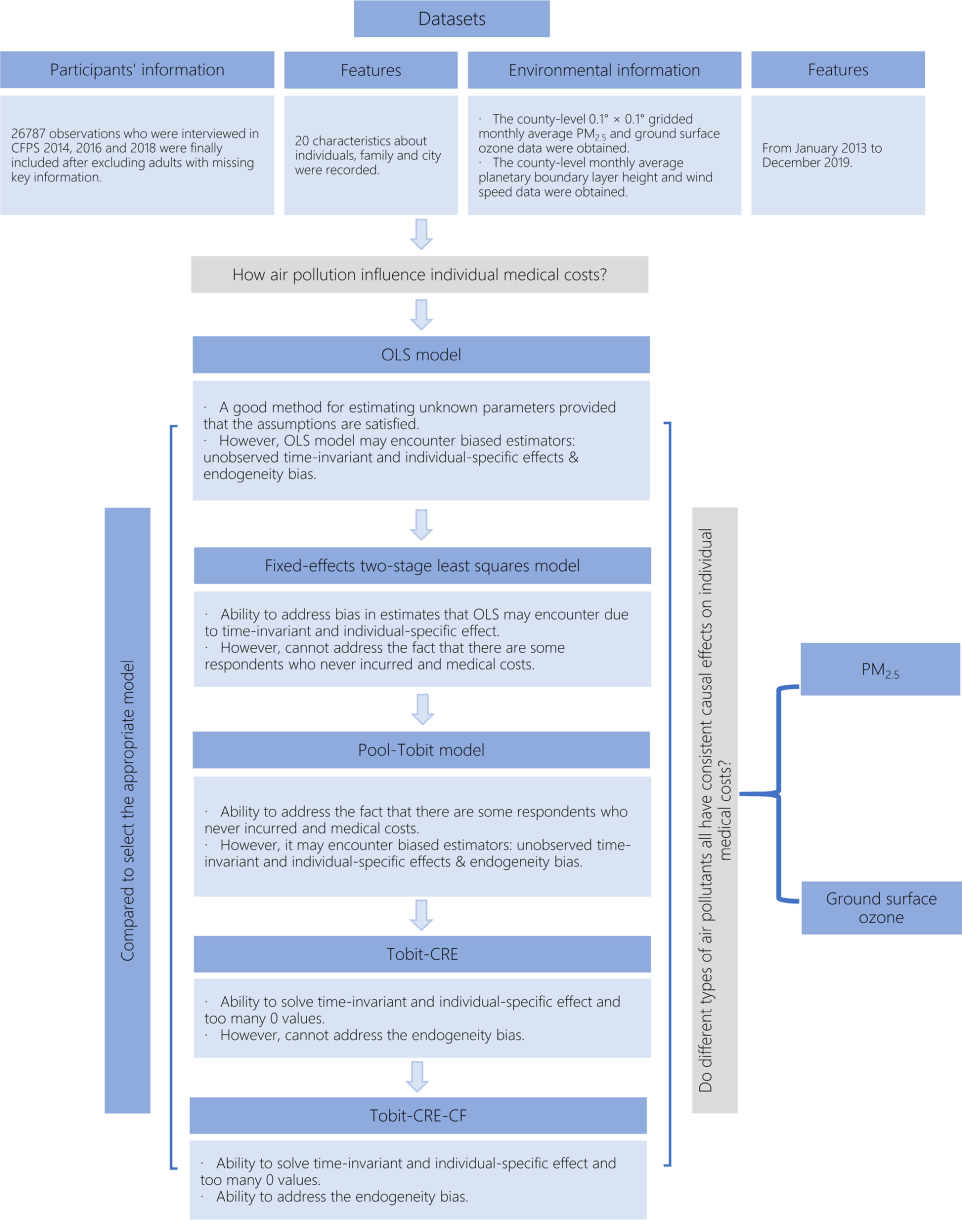
Flow chart of the participant selection and analysis

The characteristics of the participants included in this study were also compared with those of the complete 2014 survey sample, as shown in Fig. 1. Although we were not able to include all respondents due to missing data and lost follow-up, and the results of the comparison between the two groups exhibit differences in some characteristics, the characteristics of the participants in this study did not deviate particularly from the complete population. A flow chart of the participant selection and analysis process is given in Fig. 2.

### Air pollution and instrumental variables assessment

PM_2.5_ and ground surface ozone were selected as the proxy variables for air pollution in this study to explore the causal effects of different types of air pollution on medical costs. PM_2.5_ is one of the most concerning pollutants globally, producing one of the largest health burdens [5], while in China, ground surface ozone has received increased attention in recent years [16]. Another reason we chose these two pollutants is that the former represents those pollutants for which people may take conscious steps to avoid high levels of pollution, while the latter is the opposite. We will further elaborate on this point in the discussion. Monthly PM_2.5_ and ground surface ozone concentrations in China over the study period, at a 1 km × 1 km resolution, were obtained from the Tracking Air Pollution in China Database [17]. This database has been shown to have good long-term accuracy within the time frame of this study [17].

Planetary boundary layer height (PBLH) and wind speed were selected as instrumental variables in this study. PBLH reflects the depth of air at the Earth’s surface. Above the planetary boundary layer is the free atmosphere, and the transport of pollutants from the boundary layer to the free atmosphere is slow [18, 19]. Wind speed reflects the speed of air moving at a height of 10 m above the Earth’s surface. Wind speed may affect pollutant mobility and thus pollutant concentrations, while PBLH changes the concentration by changing the volume of local pollutants [20, 21]. It is generally accepted that air pollution provides the only satisfactory pathway through which wind speed and PBLH can affect health outcomes such as medical costs for residents of a specific area [20, 21]. The inclusion of two or more instrumental variables provides the opportunity to test the necessary hypotheses that make causal inferences valid [10, 11].

The ERA5 database, a global atmospheric reanalysis product with high spatial and temporal resolution, developed by the European Centre for Medium-Range Weather Forecasts (ECMWF), was accessed to obtain monthly PBLH data in China at a 2.5 km × 2.5 km resolution [22]. PBLH was measured in meters (m). The ERA5-Land was accessed to obtain monthly wind speed data in China at a resolution of 1 km × 1 km [23]. Wind speed was measured in meters per second (m/s).

In this study, participants were interviewed for three waves in 2014, 2016, and 2018, and we calculated the average PM_2.5_, ground surface ozone, PBLH, and wind speed exposure for each respondent for the twelve months prior to his/her specific interview time (to the exact month) for each wave. This allowed for a more accurate assessment of each respondent's instrumental variable data and long-term air pollution exposure.

### Medical costs assessment

The CFPS recorded the total hospitalization costs (hospitalization was defined as admission to a hospital room for at least one night due to illness or accidental injury) in the year prior to the interview; this included all medical-related and non-medical-related costs, such as medical examinations, medical treatments, accommodation, nursing care, and transportation fees due to hospitalization, regardless of whether the individual was reimbursed by health insurance. Non-hospitalization costs due to injury or illness, i.e., costs not related to the act of hospitalization in the year prior to the interview, were also recorded, including expenses such as buying medications. Thus, the total medical costs variable was recorded as the sum of the hospitalization-related and non-hospitalization costs, and this served as the dependent variable in this study.

### Covariates

The CFPS employs rigorous instruments to ensure data quality, including the use of uniformly trained surveyors, field verification and audio confirmation. The reliability of the questionnaire has been previously confirmed.

Considering that individual medical costs may be associated with a wide range of factors, a range of demographic, health-related, socioeconomic, and behavioral confounders were included as variables in this study. The demographic confounders included the age at interview and the gender of the participant. Health-related confounders included the number of chronic diseases suffered, depressive symptoms, self-rated health status, subjective memory impairment, and whether the participant was hospitalized in the last year. Socioeconomic confounders included the participant’s medical insurance, marriage status, type of household fuel, type of house, whether they obtained a subsidy, work status, and household income. In addition, the number of hospital beds per capita and income per capita in the participant’s city were recorded. Habits and behavioral confounders included drinking, smoking, exercising habits, and whether the participant surfed the internet. A set of year dummy variables was also included to control for annual unobservable fixed effects. Detailed information about the covariates is presented in Additional file 1: Text. S1.

### Tobit regression model combined with correlated random effects and control function method (Tobit-CRE-CF)

The dependent variable was the total medical costs of the participants. This was a continuous variable with a value range greater than or equal to zero. If the distribution of the dependent variable is normal or approaches normal, the linear regression model (or ordinary least squares; OLS) can be employed to explore the effects of long-term exposure to air pollution on individual medical costs. However, there are many people in China who do not spend any medical costs for a significant period of time. For example, in this study, more than 30% of participants did not spend any medical costs during the entire year prior to their interview. Thus, linear regression was not applicable in this study. As such, a two-step process was utilized to explore the effect of long-term exposure to air pollution on individual medical costs: (1) estimate whether the individual could spend medical costs first and (2) estimate the fees of the individuals who could spend medical costs. This type of response variable is also called a corner solution response or corner solution outcome [24].

The Tobit regression model is appropriate when the observed range of the dependent variable is censored in some way, as in the case of the dependent variable in the current study [11, 25]. The Tobit model modifies the likelihood function so that it reflects the unequal sampling probability of each observation, depending on whether the latent dependent variable falls above or below a defined threshold [26].

In this study, because the dependent variable, the total medical costs of the respondent in the previous year, was greater than or equal to 0, it can be assumed to be censored on the left side, with a censored value of 0, and unlimited on the right side. The Tobit model in this study was designed as follows:

1 $$y_{it}=\left\{\begin{array}{cc}y_{it}^\ast&ify_{it}^\ast>0\\0&ify_{it}^\ast\leqslant0\end{array}\right.$$

where *i* denotes the individual; *t* is the time (year); y^*^ is the latent measure of the individual total medical costs; ***X*** is the group of independent variables, including the proxy of air pollutants (PM_2.5_ and ground surface ozone), then key independent variables, and a set of covariates (also called confounders); $\beta$ is the coefficient to be estimated; $\mu$ is a random effect term.

Maximum likelihood estimation (MLE) was used for the Tobit regression model. The estimation process can be summarized in three steps: (1) obtain the probability density function for *y* in Eq. (1); (2) obtain the likelihood function based on the probability density function; (3) obtain the parameters using the Newton–Raphson method to maximize the value of the likelihood function [27, 28]. A detailed description of the Tobit regression estimation method is provided in Additional file 1: Text. S2.

The Tobit regression model is a useful benchmark but can be biased by unobserved time-invariant effects, individual-specific effects, and endogeneity [29]. Based on the relevant literature [10, 11, 24, 30], the Tobit regression model combined with correlated random effects (CRE) was employed to avoid the estimated bias caused by unobserved time-invariant and individual-specific effects, and the control function (CF) was employed to avoid endogeneity bias.

The key assumption of the CRE regression model is that the unobserved time-invariant and individual-specific effects can be denoted by the linear combination of independent variables [29]. Unobserved time-invariant and individual-specific effects can be avoided by controlling the linear combination of independent variables in the regression model. The control function (CF) can correct endogeneity problems by modeling the endogeneity in the residual. The Tobit regression model combined with CRE and CF is, thus, robust to the bias caused by unobserved time-invariant and individual-specific effects in panel data and to potential endogeneity [10, 31]. The model in this study can be described as follows:

2 $$\begin{array}{c}{Air pollutants}_{it}=g\left(Z,X\right)+{\eta }_{1}{\overline{Air pollutants} }_{i}+V\\ where:g\left(Z,X\right)={\beta }_{0}+{{{\varvec{Z}}}^{{\varvec{T}}}}_{{\varvec{i}}{\varvec{t}}}\boldsymbol{\alpha }+{{{\varvec{X}}}_{{\varvec{i}}{\varvec{t}}}}^{{\varvec{T}}}{\varvec{\theta}}++{{\overline{{\varvec{Z}}} }_{{\varvec{i}}}}^{{\varvec{T}}}{\varvec{\tau}}+{{\overline{{\varvec{X}}} }_{i}}^{{\varvec{T}}}{\varvec{\omega}}\end{array}$$

3 $$\begin{array}{c}{{y}^{*}}_{it}={\gamma }_{0}+{\gamma }_{1}{Air pollutants}_{it}+\xi V{+{\varvec{X}}}^{{\varvec{T}}}{\varvec{\theta}}{+{\mu }_{i}+\epsilon }_{it}\\ where:{\mu }_{i}=\alpha +{\varphi }_{1}{\overline{Air pollutants} }_{i}+{{\overline{{\varvec{Z}}} }_{{\varvec{i}}{\varvec{t}}}}^{{\varvec{T}}}+{{\overline{{\varvec{X}}} }_{i}}^{{\varvec{T}}}{\varvec{\rho}}+{\vartheta }_{i}\end{array}$$

where *Air pollutants* denotes the PM_2.5_ concentration and ground surface ozone concentration; ***Z*** represents the instrumental variables; ***X*** is a set of covariates; ***V*** is the residuals of the endogenous variables fitted by formula (6); y^*^ is the latent measure of the respondent’s total medical costs, as described in formula (1); ${\mu }_{i}$ is the unobserved time-invariant and individual-specific fixed effects, which can be explained by a linear combination of endogeneity variables (${\overline{Air pollutants} }_{i}$), instrumental variables (${{\overline{{\varvec{Z}}} }_{{\varvec{i}}}}^{{\varvec{T}}}$) and a set of covariates (${{\overline{{\varvec{X}}} }_{i}}^{{\varvec{T}}})$; and ${\epsilon }_{it}$ and ${\vartheta }_{i}$ are the random disturbance items. A detailed description of CRE and CF is presented in Additional file 1: Text. S2.

CRE-CF estimations are robust to endogeneity and unbiased only if the instrumental variables (***Z***) can adequately explain variations in air pollution exposure (relevant prerequisite) and lack the ability to independently explain variations in medical costs (valid prerequisite) [32]. The Cragg-Donald Wald F test and Sargan test were used to test the relevant prerequisite [33, 34] and the valid prerequisite [35], respectively. Explanations of the Cragg-Donald Wald *F* test and Sargan test are provided in Test S2 in the Supplementary File.

To test whether there were unobserved time-invariant effects, individual-specific effects and potential endogeneity, the Hausman specification test was employed in this study with the null hypothesis that the differences in the estimates are not systematic [36]. A detailed description of the Hausman specification tests used in this study is provided in Test S2 in the Supplementary File.

All data management was performed using R 4.0.2. The statistical analyses were performed with Stata (Version 15.0 SE, Stata Crop, Chicago, IL, USA). *P* < 0.05 was used to determine statistical significance. The standard errors of all estimations in the CRE-CF regression model were computed by the bootstrap method through the resampling of the dataset 100 times to obtain more robust estimates.

## Results

### Descriptive results

Descriptive statistics for the categorical and continuous variables are reported in Table 1. Columns (1)–(4) represent 2014, 2016, 2018, and the full sample, respectively. Due to space limitations, the text mainly presents the descriptive statistics of variables in the full sample; the details for 2014, 2016, and 2018 are shown in Table 1.

**Table 1:** Descriptive statistics for the key variables in this study

	**(1)**	**(2)**	**(3)**	**(4)**
**Variables**	**2014**	**2016**	**2018**	**Total**
Total medical costs (*RMB*)	2347.16 (7908.26)	3045.24 (12,337.98)	3958.84 (13,829.97)	3117.08 (11,652.05)
Average PM_2.5_ concentrations (*µg/m*^*3*^)	56.45 (22.06)	47.98 (17.86)	41.05 (13.56)	48.49 (19.22)
Ground surface ozone (*µg/m*^*3*^)	105.71 (9.81)	111.19 (11.87)	121.11 (15.21)	112.67 (14.02)
Planetary boundary layer height (*m*)	489.26 (68.79)	487.53 (74.86)	487.68 (70.39)	488.16 (71.39)
Wind speed (*m/s*)	1.03 (0.55)	1.00 (0.50)	1.07 (0.57)	1.03 (0.54)
Age (y*ear*)	50.11 (13.00)	52.16 (12.98)	54.11 (13.00)	52.13 (13.09)
Gender				
Female	4627 (51.83)	4627 (51.83)	4627 (51.83)	13,881 (51.83)
Male	4301 (48.17)	4301 (48.17)	4301 (48.17)	12,903 (48.17)
Number of chronic diseases				
Zero	7083 (79.33)	7082 (79.32)	6922 (77.53)	21,087 (78.73)
One	1160 (12.99)	1086 (12.16)	1050 (11.76)	3296 (12.31)
Two or more	685 (7.67)	760 (8.51)	956 (10.71)	2401 (8.96)
CES-D scores	9.06 (3.83)	32.27 (8.00)	33.10 (8.20)	24.81 (13.14)
Whether healthier than last year				
Healthier	850 (9.52)	800 (8.96)	861 (9.64)	2511 (9.38)
No change	5085 (56.96)	4984 (55.82)	4631 (51.87)	14,700 (54.88)
Worse	2993 (33.52)	3144 (35.22)	3436 (38.49)	9573 (35.74)
Whether hospitalized last year				
No	7905 (88.54)	7712 (86.38)	7509 (84.11)	23,126 (86.34)
Yes	1023 (11.46)	1216 (13.62)	1419 (15.89)	3659 (13.66)
Subjective memory impairment				
Very bad	2139 (23.96)	1506 (16.87)	1760 (19.71)	5405 (20.18)
Bad	2253 (25.24)	2304 (25.81)	1404 (15.73)	5961 (22.26)
Not bad	2309 (25.86)	2619 (29.33)	2707 (30.32)	7635 (28.51)
Well	1220 (13.66)	1422 (15.93)	1831 (20.51)	4473 (16.70)
Very well	1007 (11.28)	1077 (12.06)	1226 (13.73)	3310 (12.36)
Medical insurance				
UEBMI	1372 (15.37)	1419 (15.89)	1432 (16.04)	4223 (15.77)
URBMI	716 (8.02)	700 (7.84)	801 (8.97)	2217 (8.28)
NCMS	6575 (73.64)	6604 (73.97)	6492 (72.72)	19,671 (73.44)
Others	265 (2.97)	205 (2.30)	203 (2.27)	673 (2.51)
Marriage status				
Married	8091 (90.63)	8079 (90.49)	7987 (89.46)	24,157 (90.19)
Single	327 (3.66)	244 (2.73)	212 (2.37)	783 (2.92)
Divorce/widowed	510 (5.71)	605 6.78)	729 (8.17)	1844 (6.88)
Household cooking fuel				
Clean	5283 (59.17)	5658 (63.37)	6195 (69.39)	17,139 (63.98)
Unclean	3645 (40.83)	3270 (36.63)	2733 (30.61)	9648 (36.02)
Type of house				
One-story	4089 (45.80)	3785 (42.39)	4010 (44.91)	11,884 (44.37)
Multi-story	4312 (48.30)	4491 (50.30)	3844 (43.06)	12,647 (47.22)
Unknown	527 (5.90)	652 (7.30)	1074 (12.03)	2253 (8.41)
Whether obtaining any subsidy				
Yes	3646(40.84)	4365 (48.89)	4424 (49.55)	12,435 (46.43)
No	5282 (59.16)	4563 (51.11)	4504 (50.45)	14,349 (53.57)
Work status				
Unemployed	55 (0.62)	50 (0.56)	45 (0.50)	150 (0.56)
Employed	6997 (78.37)	6867 (76.92)	6730 (75.38)	20,594 (76.89)
Retired	1838 (20.59)	1995 (22.35)	2149 (24.07)	5982 (22.33)
Unknown	38 (0.43)	16 (0.18)	4 (0.04)	58 (0.22)
Habit of surfing the internet				
Yes	1857 (20.80)	2730 (30.58)	3681 (41.23)	8268 (30.87)
No	7071 (79.20)	6198 (69.42)	5247 (58.77)	18,516 (69.13)
Habit of drinking				
No	7487 (83.86)	7527 (84.31)	7493 (83.93)	22,507 (84.03)
Yes	1441 (16.14)	1401 (15.69)	1435 (16.07)	4277 (15.97)
Whether smoking				
No	6266 (70.18)	6356 (71.19)	6294 (70.50)	18,916 (70.62)
Yes	2662 (29.82)	2572 (28.81)	2634 (29.50)	7868 (29.38)
Exercise habits				
Frequently	1804 (20.21)	2371 (26.56)	2871 (32.16)	7046 (26.31)
Occasionally	1217 (13.63)	1267 (14.19)	1583 (17.73)	4067 (15.18)
Hardly/never	5907 (66.16)	5290 (59.25)	4474 (50.11)	15,671 (58.51)
Household per capita income quantile				
0–25%,1st	2022 (22.65)	2331 (26.11)	2275 (25.48)	6628 (24.75)
25–50%, 2nd	2158 (24.17)	2517 (28.19)	2591 (29.02)	7266 (27.13)
50–75%, 3rd	2335 (26.15)	2303 (25.80)	2308 (25.85)	6946 (25.93)
75–100%, 4th	2413 (27.03)	1777 (19.90)	1754 (19.65)	5944 (22.19)
City per capita income	50,108.94 (14,143.32)	61,082.38 (18,472.41)	73,816.49 (22,000.81)	61,669.27 (20,870.87)
City per capita hospital beds	47.40 (18.27)	51.20 (19.07)	49.49 (20.40)	49.36 (19.33)
Total	8928	8928	8928	26,784

*Note*: (1) For the continuous, statistics reported are the sample mean with the standard deviation in parentheses; (2) For the categorical, statistics reported are the sample frequency with the percentage in parentheses; (3) *UEBMI* Urban Employment Basic Medical Insurance, *URBMI* Urban Residents Basic Medical Insurance, *NCMS* New Cooperative Medical Scheme, Others free medical care, another supplement insurance, and full-self fee

As shown in Table 1, the full sample included 8928 participants with a total of 26,784 observations, and the average age of the participants was approximately 50 years. The average total medical costs were 3117.08 RMB. The average PM_2.5_ concentration, ground surface ozone, PBLH, and wind speed were 48.49 µg/m^3^, 112.67 µg/m^3^, 488.16 m, and 1.03 m/s, respectively. There were slightly more females (51.83%) than males (48.17%). Nearly 80% of the participants across all waves of the study (78.73%) did not suffer from any chronic disease, and only approximately 9% suffered from two or more chronic diseases. The average CES-D score was 24.81, and approximately 55% of participants across all waves had no change in self-rated health condition compared to the previous year. Across all waves, nearly 87% of the participants were not hospitalized in the year prior to the interview.

Across all waves, more than 90% were married. More than 60% (63.98%) of participants across all waves used clean cooking fuel in their daily life. Overall, 44.37% of participants lived in a one-story house during the study period, while 47.22% lived in a multi-story house. More than 50% did not receive any government subsidies. Across all waves, a total of 76.89% of participants were employed and nearly 70% did not report surfing the internet. Nearly 85% did not drink alcohol more than three times a week, and more than 80% did not smoke in daily life. Nearly 60% hardly or never take exercise in daily life.

### Regression results

Tables 2 and 3 display the impact of exposure to PM_2.5_ and ground surface ozone on individual total medical costs, respectively. Columns (1)–(5) of the tables report the regression results for the baseline linear (OLS), fixed-effects combined with two-stage least square (FE-2SLS), Pool-Tobit, Tobit-CRE, and Tobit-CRE-CF models, respectively. For each model, the variable marginal effects are reported. Moreover, the results of the Hausman test, Cragg-Donald Wald *F* statistic, and Sargan statistical tests are displayed in the last three rows of Tables 2 and 3. Due to space limitations, only the estimated coefficients of the main explanatory variables of interest and the results of some tests are displayed in Tables 2 and 3. The estimations for the other covariates are shown in Additional file 1: Table S1-S2.

**Table 2:** Estimation results of the effects of PM_2.5_ on the total medical costs

Variables	ln (total medical costs)		Total medical costs		
	(1)	(2)	(3)	(4)	(5)
	Linear regression	Fixed-effects 2SLS	Pool-Tobit	Tobit-CRE	Tobit-CRE-CF
PM_2.5_	− 0.008***	0.052	− 16.527***	− 47.180*	526.396*
	(0.001)	(0.048)	(4.598)	(18.675)	(232.711)
PM_2.5__residual	-	-	-	-	− 576.490*
	-	-	-	-	(230.521)
Other control variables	Yes	Yes	Yes	Yes	Yes
Year dummy	Yes	Yes	Yes	Yes	Yes
Correlated random effects	No	No	No	Yes	Yes
*N*	26,784	26,784	26,784	26,784	26,784
*R*^*2*^	0.342	0.180	-	-	-
Cragg-Donald Wald *F* statistic	-	48.229 > 19.93	-	-	-
Sargan statistic	-	1.245 (*P* = 0.265 > 0.05)	-	-	-

*Note*: (1) Standard errors in parentheses; (2) Other control variables include a set of covariates; (3) Correlated random effects: a linear combination of endogenous variables, instrumental variables and covariates; (4) *p < 0.05, **p < 0.01, ***p < 0.001

**Table 3:** Estimation results of the effects of ground surface ozone on the total medical costs

Variables	ln (total medical fees)		Total medical fees		
	(1)	(2)	(3)	(4)	(5)
	Linear regression	Fixed-effects 2SLS	Pool-Tobit	Tobit-CRE	Tobit-CRE-CF
Ground surface ozone	− 0.009***	0.023	− 4.859	46.420**	198.626*
	(0.001)	(0.019)	(6.752)	(16.254)	(87.428)
Ozone_residual	-	-	-	-	− 156.447
	-	-	-	-	(91.514)
Other control variables	Yes	Yes	Yes	Yes	Yes
Year dummy	Yes	Yes	Yes	Yes	Yes
Correlated random effects	No	No	No	Yes	Yes
*N*	26,784	26,784	26,784	26,784	26,784
*R*^*2*^	0.341	0.192	-	-	-
Cragg-Donald Wald *F* statistic		241.401 > 19.93	-	-	-
Sargan statistic		0.898(*P* = 0.343 > 0.05)	-	-	-

*Note*: (1) Standard errors in parentheses; (2) Other control variables include a set of covariates; (3) Correlated random effects: a linear combination of endogenous variables, instrumental variables and covariates; (4) *p < 0.05, **p < 0.01, ***p < 0.001

The *P* values of the Hausman specification tests between the estimates of the Pool-Tobit and Tobit-CRE regression models were smaller than 0.05, suggesting the presence of unobserved time-invariant and individual-specific effects that could cause bias in the estimation of the Tobit regression model. In addition, since the estimation of the residuals of PM_2.5_ was statistically significant, it is likely that the endogeneity of PM_2.5_ would cause bias, and the resulting estimation results of the Tobit-CRE model would be inconsistent. The FE-2SLS model does not consider the distribution of the total medical costs, in which more than 30% of observations were equal to 0; thus, this would lead to biased estimates. Considering the inconsistent estimations provided by the OLS, FE-2SLS, Pool-Tobit, and Tobit-CRE regression models, the following discussions are mainly based on the results estimated by the Tobit-CRE-CF regression model.

The coefficient of PM_2.5_ estimated by the Tobit-CRE-CF regression model was 526.396, as shown in column (5) of Table 2, and it was statistically significant at the 5% level, indicating that PM_2.5_ exposure concentration was positively associated with total medical costs. This suggests that long-term exposure to PM_2.5_ increases CFPS participants’ total medical costs. However, the PM_2.5_ coefficients estimated by the OLS, FE-2SLS, Pool-Tobit, and Tobit-CRE models were − 0.008, 0.052, − 16.527, and − 47.180, as shown in columns (1)–(4) of Table 2, respectively. Some of these values contrast with the estimate provided by the Tobit-CRE-CF regression model, suggesting that neglecting the dependent variable distribution and the presence of unobserved time-invariant and individual-specific fixed effects as well as potential endogeneity cause substantial biases and can even lead to the wrong conclusion.

The coefficient of ground surface ozone estimated by the Tobit-CRE-CF regression model was 198.626, as shown in column (4) of Table 3, and it was statistically significant at the 5% level. This indicates that ground surface ozone exposure is also positively associated with total medical costs. However, the coefficients for ground surface ozone estimated by the OLS, FE-2SLS, Pool-Tobit, and Tobit-CRE regression models were − 0.009, − 0.023, − 4.859, and − 46.420, respectively, as shown in columns (1)–(4) of Table 3. Some of these values are inconsistent with the estimate provided by the Tobit-CRE-CF regression model. Again, this indicates that neglecting the dependent variable distribution and the presence of unobserved time-invariant and individual-specific fixed effects, as well as potential endogeneity, cause significant bias. This highlights the importance of the empirical strategies employed in the current study.

For PM_2.5_ and ground surface ozone, the Cragg-Donald Wald *F* statistics were both greater than the Stock-Yogo weak ID test critical value of 10%, suggesting that the relevant prerequisite was valid. Namely, the instrumental variables are both strongly related to the endogenous variable. The *P* values of the Sargan statistics were both greater than 0.05, indicating that overidentification did not exist in this study. This indirectly suggests that the valid prerequisite of the instrumental variables was met.

In this study, the Tobit-CRE-CF regression model, a kind of limited dependent model, is used to estimate more accurate effects. However, as a compromise, it is hard to interpret the coefficients. To understand the results better, we calculate the marginal effect of the model: (1) the marginal effects for the latent measure of total medical costs (y^*^); (2) the marginal effects for the latent measure of total medical costs in the condition where the observed total medical costs were greater than 0; namely, the marginal effects for the latent measure of total medical costs for the individuals who could spend medical costs last year (y^*^|y > *0*); and (3) the marginal effects for the observed measure of total medical costs in the condition where the observed total medical costs were equal to 0; namely, the marginal effects for the observed measure of total medical costs for the individuals who could not spend medical costs last year (*y|y* > *0*). Specifically, for PM_2.5_ concentration and ground surface ozone, the margin effects for the observed measure of the total medical costs for the individuals who spent any medical fees last year (*y*|*y* > 0) are 199.144 and 75.145, respectively, and both significant at 5% level, which means that when PM_2.5_ concentration and ground surface ozone increase one unit, the total medical costs for the individuals who spent medical fee last year increase by 199.144 and 75.145 RMB, respectively. The results are shown in Additional file 1: Table S3.

### Modification analysis

We preliminarily explored whether the effects of long-term air pollution exposure on individual medical costs are mitigated by gender and age, and the estimates are shown in Additional file 1: Table S4.

Column (1) and (2) of Table S4 show the results of the modification analysis of gender for PM_2.5_ concentration and ground surface ozone, and column (3) and (4) show the results of the modification analysis of age for PM_2.5_ and ground surface ozone. The estimated coefficient of the interaction term between female and PM_2.5_ is greater than 0 and significant at 5% level, which means the harmful effects of long-term PM_2.5_ exposure on individual medical costs are greater than male, while the modification effects of gender are not significant for ground surface ozone exposure. The estimated coefficient of the interaction term between age and ground surface ozone is greater than 0 and significant at 5% level, which means that the order the individual, the greater the harmful effects of long-term ground surface ozone exposure on the individual medical costs, while the modification effects of age are not significant for PM_2.5_ exposure.

### Robustness test

Two strategies were adopted to exclude the effects of extreme total medical cost values. First, the total medical cost data were trimmed so that maximum costs of 1% were deleted. Using the same regression model and methods, the robustness of our findings was tested. Second, the upper limit of the total medical costs was set to 100,000 RMB in the Tobit-CRE-CF. In this case, if the total medical costs were more than 100,000 RMB, they were recorded as 100,000 RMB.

Columns (1) and (2) of Table 4 report the results of the robustness test of the trimmed 1% dataset. The PM_2.5_ and ground surface ozone coefficients were 198.836 and 72.748, respectively, and both were statistically significant at the 5% level. Columns (3) and (4) of Table 4 report the results of the robustness test based on the Tobit-CRE-CF model with an upper limit of 100,000 RMB. The PM_2.5_ and ground surface ozone coefficients were 484.622 and 175.289, respectively, and both were statistically significant at the 5% level. These results indicate that the PM_2.5_ concentration and ground surface ozone exposure concentration are positively associated with total medical costs, which is consistent with the main regression analyses. This suggests that the results are robust.

**Table 4:** The estimated results of robust test

Variables	Total medical fees					
	Trimmed 1%		Up limit 100,000 RMB		Non-hospitalization medical costs	
	(1)	(2)	(3)	(4)	(5)	(6)
PM_2.5_	198.836*	-	484.622*	-	238.334*	-
	(85.367)	-	(188.291)	-	(93.762)	-
PM_2.5__residual	− 220.51*	-	− 528.834**	-	− 258.637**	-
	(87.291)	-	(187.738)	-	(93.031)	-
Ground surface ozone	-	72.748*	-	175.289*	-	92.122*
	-	(36.066)	-	(73.091)	-	(35.984)
Ozone_residual	-	− 64.072	-	− 141.464	-	− 80.904*
	-	(38.052)	-	(76.652)	-	(36.320)
Other control variables	Yes	Yes	Yes	Yes	Yes	Yes
Year dummy	Yes	Yes	Yes	Yes	Yes	Yes
Correlated random effects	Yes	Yes	Yes	Yes	Yes	Yes
*N*	26,133	26,133	26,784	26,784	26,784	26,784

*Note*: (1) Standard errors in parentheses; (2) Other control variables include a set of covariates; (3) Correlated random effects: a linear combination of endogenous variables, instrumental variables and covariates; (4) *p < 0.05, **p < 0.01, ***p < 0.001

Non-hospitalization medical costs were also used as a dependent variable to test whether the causal effect of air pollution on medical costs persisted after excluding hospitalization costs. Columns (5) and (6) of Table 4 report the results of this robustness test. The PM_2.5_ and ground surface ozone coefficients were 238.334 and 92.122, respectively, and both were statistically significant at the 5% level. These results indicate that the PM_2.5_ concentration and ground surface ozone exposure are positively associated with non-hospitalization medical costs, suggesting that the causal effect of air pollution on medical costs persists after excluding hospitalization costs. Only the estimated coefficients of the independent variables of interest and the results of some tests are displayed in Table 4.

## Discussion

This study successfully utilized a large representative national longitudinal cohort study to construct causal models using the Tobit-CRE-CF method and found that long-term exposure to air pollutants may lead to higher medical costs for individuals, regardless of whether the air pollution is severe enough to cause an individual to be hospitalized and whether the individual may take different measures to cope with the air pollutants.

This study has the following strengths. First, to our knowledge, it is the first to assess the effects of different types of air pollutants on individual healthcare costs over time using a large, long-term, national cohort, and corresponding causal models. Currently, the literature mainly focuses on the health impact of air pollution and lacks evidence from developing countries [37], individual-level evidence [9, 38], and comparisons of different types of pollutants [39]. Moreover, most published studies do not consider causal effects [40] or the reality that a significant portion of the population may not spend healthcare costs for a long period of time. This is particularly important when exploring economic burdens, as an accurate assessment of the impacts of air pollution can be decisive in how policymakers adopt relevant measures [13].

Importantly, as mentioned above, this study provides a useful response to the problem of a significant proportion of respondents with zero medical costs incurred over the study period. China began its healthcare reform in 2009 in response to public dissatisfaction with the limited accessibility and high cost of healthcare, and while considerable results have been achieved, more progress is expected [41]. Thus, among the respondents who did not spend any medical expenses in the year prior to their interview, while some of them may have been in good health and did not need to spend any medical costs, others may have needed to seek medical help but did not due to economic conditions, the accessibility of medical services, and other reasons [41, 42]. It is not reasonable to generalize these respondents to the group with zero medical costs and directly perform OLS or IV + 2SLS [24]. Therefore, in this paper, a Tobit regression model was used to determine whether the respondents were expected to spend medical costs based on their comprehensive characteristics. This effectively prevented the bias caused by the mishandling of too many 0 values.

In addition, this study developed, applied, and validated the Tobit-CRE-CF method, which is able to deal simultaneously with time-invariant effects, individual-specific effects, endogeneity of air pollutants, and the presence of some participants who had not to spend any medical costs during the study period [10, 11, 24, 43]. In previous explorations of the relationship between air pollutant exposure and adverse health outcomes, the biggest obstacle often encountered is the endogeneity of air pollution [12, 30, 44]. One commonly cited explanation is reverse causality, i.e., endogeneity due to the possibility that people with high medical costs may engage in air pollution mitigation behaviors such as “chasing clean air” [45]. The results of the OLS, Pool-Tobit, and Tobit-CRE models in this study suggested that long-term exposure to air pollutants may contribute to lower healthcare costs even after comprehensively controlling for confounding factors. This result largely corroborates the presence of endogeneity of air pollution. Based on this consideration, the endogeneity of air pollution was controlled using the CF method. We were fortunate to identify two reasonable instrumental variables, PBLH, and wind speed, and their rationality and validity were demonstrated by a series of rigorous tests. These results verified the validity of the observed causal effects.

Long-term exposure to air pollutants may have multiple adverse effects on human health. PM_2.5_ exposure may cause asthma [46], atherosclerosis [47], etc., while ground surface ozone may cause cardiovascular disease [48], emphysema [49], and damage to the central nervous system [50], etc., all of which may lead to higher individual medical costs. This study explored the causal effects of different types of pollutants on medical costs using PM_2.5_ and ground surface ozone as two representatives. The consideration is that people may have different responses to different pollutants; for example, most people are more familiar with PM_2.5_ pollution, which is usually widely publicized and one of the pollutants that causes the greatest adverse health and economic burden, and are therefore more likely to take appropriate protective measures, such as reducing exposure by going out less or wearing a mask [15, 51], whereas they are less familiar with ground surface ozone pollution, which although it has gained increasing attention in recent years in the Chinese government and often has difficulty judging its severity, they are more likely to go out in brighter weather when ozone pollution is more severe, increasing exposure [7, 14, 52]. The importance of controlling air pollutants is further supported by the finding in this study that long-term exposure to different types of air pollutants causes an increase in individual medical costs.

The medical costs in this study evaluated the sum of total hospitalization medical costs and total non-hospitalization medical costs of respondents due to illness or accidental injury. It is worth noting that the costs evaluated include not only direct medical costs, such as medicine, treatment, and ward fees but also indirect medical costs, such as lodging, meals, and caregiver fees. Considering the rigorous instruments adopted by the CFPS to ensure data quality, this gives us the opportunity to comprehensively evaluate the complete economic impact of air pollution exposure. In addition, this study further validates that there is a consistent effect of air pollution exposure on the costs associated with medical actions that are not severe enough to warrant hospitalization.

Despite its strengths, there are several limitations of this study that should be noted. First, the pollutant exposure estimates in this study were made at the district/county level, which requires the assumption that respondents always stay within their district/county. However, given that only those who did not move out of the district/county during the study period were included, this is not likely to have had a substantial impact on the study conclusions. Second, despite the detailed justification of the research process in this paper, caution should be adopted when inferring the causal impact of long-term air pollution on individual medical costs, and future studies should combine multiple causal approaches to comprehensively assess the causal effects of air pollutants on medical costs while conducting a full range of heterogeneity and mechanism exploration. Third, it is possible that some minor diseases or injuries caused by long-term exposure to air pollutants would not prompt individuals to seek help and spend costs, regardless of whether they are hospitalization-related or non-hospitalization-related medical costs. In addition, data limitations make it difficult to assess the costs associated with death, which should have been included in medical cost considerations. Nonetheless, if this was the case, the current estimates would provide an underestimation of the effects of long-term exposure to air pollutants on individual medical costs. Finally, the rigorous methodology adapted in this paper to more accurately assess the causality of air pollution on health care costs also allows us to make some concessions in the ability to fully assess health care costs in China. Further research should be conducted in the future to assess the economic burden associated with air pollution.

## Conclusions

Using a large, long-term, representative nationwide cohort, this study developed and validated a Tobit-CRE-CF model to control for unobserved time-invariant effects, individual-specific effects, and endogeneity and to address the issue of the large number of participants who did not spend medical costs during the study period. The results revealed that long-term exposure to air pollutants contributes to higher individual medical costs, regardless of whether the air pollution causes individuals to get sick enough to require hospitalization or whether individuals may adopt various measures to minimize their pollutant exposure.

## Supplementary Information

**Additional file 1:** **Text S1.** Description of covariates. **Text S2.** Details of Tobit-CRE-CF. **Figure S1.** Coverage, number of participants, and pollutant concentrations in surveyed counties/districts across China in 2014. **Figure S2.** Coverage, number of participants, and pollutant concentrations in surveyed counties/districts across China in 2016. **Figure S3.** Coverage, number of participants, and pollutant concentrations in surveyed counties/districts across China in 2018. **Table S1.** Estimation results of the effects of PM_2.5_ on the total medical costs. **Table S2.** Estimation results of the effects of ground surface ozone on the total medical costs. **Table S3.** Various margin effects for the key independent variables in the model of Tobit-CRE-CF. **Table S4.** Heterogeneity analysis.

## Data Availability

The datasets that support the findings of this study are available on the ECMWF website (https://www.ecmwf.int/en/era5-land/), TAP website (http://tapdata.org.cn/), and the CFPS website (https://www.isss.pku.edu.cn/cfps/).

## Abbreviations

CFPS: Chinese Family Panel Study
ECMWF: The European Centre for Medium-Range Weather Forecasts
ERA5: The fifth generation ECMWF atmospheric reanalysis dataset
FE-2SLS: Fixed-effects combined with two-stage least square
GDP: Gross domestic product
MLE: Maximum likelihood estimation
OLS: Ordinary least squares
PBLH: Planetary boundary layer height
Tobit-CRE-CF: Tobit model combined with a correlated random effects and control function approach

## References

[1] Grande G, Ljungman PL, Eneroth K, Bellander T, Rizzuto D (2020). Association between cardiovascular disease and long-term exposure to air pollution with the risk of dementia. JAMA Neurol.

[2] Qiu H, Schooling CM, Sun S (2018). Long-term exposure to fine particulate matter air pollution and type 2 diabetes mellitus in elderly: a cohort study in Hong Kong. Environ Int.

[3] Raaschou-Nielsen O, Andersen ZJ, Hvidberg M (2011). Lung cancer incidence and long-term exposure to air pollution from traffic. Environ Health Perspect.

[4] Altieri KE, Keen SL (2019). Public health benefits of reducing exposure to ambient fine particulate matter in South Africa. Sci Total Environ.

[5] Cai W, Zhang C, Suen HP (2021). The 2020 China report of the Lancet Countdown on health and climate change. Lancet Public Health.

[6] Lu X, Hong J, Zhang L (2018). Severe surface ozone pollution in China: a global perspective. Environ Sci Technol Lett.

[7] Lu X, Zhang S, Xing J, et al. Progress of air pollution control in China and its challenges and opportunities in the ecological civilization era. Engineering. 2020;6(12):1423-1431.

[8] Yin P, Brauer M, Cohen AJ (2020). The effect of air pollution on deaths, disease burden, and life expectancy across China and its provinces, 1990–2017: an analysis for the Global Burden of Disease Study 2017. Lancet Planet Health.

[9] Xie Y, Dai H, Dong H, Hanaoka T, Masui T (2016). Economic impacts from PM2. 5 pollution-related health effects in China: a provincial-level analysis. Environ Sci Technol.

[10] Wooldridge JM (2015). Control function methods in applied econometrics. J Human Res.

[11] Wooldridge JM. Introductory econometrics: a modern approach: Cengage learning, Boston. 2015.

[12] Xu Z, Liu Z, Lu L (2022). Assessing the causal effects of long-term exposure to PM2. 5 during pregnancy on cognitive function in the adolescence: evidence from a nationwide cohort in China. Environ Pollut.

[13] Deryugina T, Heutel G, Miller NH, Molitor D, Reif J (2019). The mortality and medical costs of air pollution: evidence from changes in wind direction. Am Econ Rev.

[14] Wang T, Xue L, Brimblecombe P, Lam YF, Li L, Zhang L (2017). Ozone pollution in China: a review of concentrations, meteorological influences, chemical precursors, and effects. Sci Total Environ.

[15] Xu J, Gao C, Lee JKW, Zhao J (2017). PM2. 5: a barrier to fitness and health promotion in China. J Sport Health Sci.

[16] Lu X, Zhang S, Xing J (2020). Progress of air pollution control in China and its challenges and opportunities in the ecological civilization era. Engineering.

[17] Geng G, Xiao Q, Liu S, et al. Tracking air pollution in china: near real-time pm2. 5 retrievals from multiple data sources. arXiv preprint arXiv:210306520. 2021. 10.1021/acs.est.1c01863 34407614

[18] Seinfeld J, Pandis S (2016). Aerosols. Atmospheric chemistry and physics: from air pollution to climate change.

[19] Finlayson-Pitts BJ, Pitts JN (1986). Atmospheric chemistry. Fundamentals and experimental techniques.

[20] Schwartz J, Fong K, Zanobetti A (2018). A national multicity analysis of the causal effect of local pollution, NO 2, and PM 2.5 on mortality. Environ Health Perspect.

[21] Schwartz J, Bind M-A, Koutrakis P (2017). Estimating causal effects of local air pollution on daily deaths: effect of low levels. Environ Health Perspect.

[22] Hersbach H, Bell B, Berrisford P (2020). The ERA5 global reanalysis. Q J R Meteorol Soc.

[23] Muñoz-Sabater J, Dutra E, Agustí-Panareda A (2021). ERA5-Land: A state-of-the-art global reanalysis dataset for land applications. Earth Syst Sci Data.

[24] Wooldridge JM. Econometric analysis of cross section and panel data. MIT Press. 2010.

[25] McDonald JF, Moffitt RA (1980). The uses of Tobit analysis. The review of economics and statistics.

[26] Kennedy P. A guide to econometrics: Wiley; 2008.

[27] Orme C (1989). On the uniqueness of the maximum likelihood estimator in truncated regression models. Econometric Rev.

[28] Grossman M (1972). On the concept of health capital and the demand for health. J Polit Economy.

[29] Joshi R, Wooldridge JM (2019). Correlated random effects models with endogenous explanatory variables and unbalanced panels. Ann Econ Stat.

[30] Ju K, Lu L, Chen T, et al. Does long-term exposure to air pollution impair physical and mental health in the middle-aged and older adults?—A causal empirical analysis based on a longitudinal nationwide cohort in China. Sci Total Environ. 2022;827:154312. 10.1016/j.scitotenv.2022.154312 35248644

[31] Petrin A, Train K (2010). A control function approach to endogeneity in consumer choice models. J Marketing Res.

[32] Burns DK, Jones AP, Goryakin Y, Suhrcke M (2017). Is foreign direct investment good for health in low and middle income countries? An instrumental variable approach. Soc Sci Med.

[33] Desbordes R, Verardi V (2012). A robust instrumental-variables estimator. Stand Genomic Sci.

[34] Sanderson E, Windmeijer F (2016). A weak instrument F-test in linear IV models with multiple endogenous variables. J Econometrics.

[35] Ullah S, Zaefarian G, Ullah F. How to use instrumental variables in addressing endogeneity? A step-by-step procedure for non-specialists. Elsevier; 2021. p. A1-A6.

[36] Hausman JA. Specification tests in econometrics. Econometrica: J Econ Soc. 1978:1251–71.

[37] Ju K, Lu L, Liao W, et al. Long-term exposure of PM2.5 components on the adults’ depressive symptoms in China – evidence from a representative longitudinal nationwide cohort. Sci Total Environ. 2022:159434. 10.1016/j.scitotenv.2022.159434 36244492

[38] Chen S, Bloom DE (2019). The macroeconomic burden of noncommunicable diseases associated with air pollution in China. PLoS ONE.

[39] Niu Y, Chen R, Kan H. Air pollution, disease burden, and health economic loss in China. Ambient Air Pollution and Health Impact in China 2017:233–42. 10.1007/978-981-10-5657-4_10 29177965

[40] Li L, Du T, Zhang C (2020). The impact of air pollution on healthcare expenditure for respiratory diseases: evidence from the People’s Republic of China. Risk Manage Healthcare Policy.

[41] Yip W, Fu H, Chen AT, Zhai T, Chen W (2019). 10 years of health-care reform in China: progress and gaps in Universal Health Coverage. Lancet.

[42] Hu H, Jian W, Fu H, Zhang H, Pan J, Yip W (2021). Health service underutilization and its associated factors for chronic diseases patients in poverty-stricken areas in China: a multilevel analysis. BMC Health Serv Res.

[43] Wooldridge JM (2011). A simple method for estimating unconditional heterogeneity distributions in correlated random effects models. Econ Letters.

[44] Ju K, Lu L, Wang W, et al. Causal effects of air pollution on mental health among adults——an exploration of susceptible populations and role of physical activity based on a longitudinal nationwide cohort in China. Environ Res 2022:114761. 10.1016/j.envres.2022.114761 36372147

[45] Chen S, Chen Y, Lei Z, Tan-Soo J-S (2021). Chasing clean air: pollution-induced travels in China. J Assoc Environ Resour Econ.

[46] Loftus C, Yost M, Sampson P (2015). Regional PM2. 5 and asthma morbidity in an agricultural community: a panel study. Environ Res.

[47] Tian M, Zhao J, Mi X (2021). Progress in research on effect of PM2. 5 on occurrence and development of atherosclerosis. J Appl Toxicol.

[48] Mazidi M, Speakman JR (2018). Impact of obesity and ozone on the association between particulate air pollution and cardiovascular disease and stroke mortality among US adults. J Am Heart Assoc.

[49] Paulin LM, Gassett AJ, Alexis NE (2020). Association of long-term ambient ozone exposure with respiratory morbidity in smokers. JAMA Intern Med.

[50] Martínez-Lazcano JC, González-Guevara E, del Carmen RM (2013). The effects of ozone exposure and associated injury mechanisms on the central nervous system. Rev Neurosci.

[51] Xiong L, Li J, Xia T (2018). Risk reduction behaviors regarding PM2. 5 exposure among outdoor exercisers in the Nanjing metropolitan area, China. Int J Environ Res Public Health.

[52] Fan Yang YP, Zeyu Zou. Patterns and deterninants pf physical activity of elderly people in China (In Chinese). China Institute Sport Sci. 2019;55(10):10-21+40.

