# Supplementary material for: Identifying the causal effects of long-term exposure to PM2.5 and ground surface ozone on individual medical costs in China—evidence from a representative longitudinal nationwide cohort
Source: BMC Med. 2023 Apr 3;21:127. doi: 10.1186/s12916-023-02839-1 (PMC10071749; doi:10.1186/s12916-023-02839-1)
Supplement: Supplementary file 1 — Additional file 1: Text S1. Description of covariates. Text S2. Details of Tobit-CRE-CF. Figure S1. Coverage, number of participants, and pollutant concentrations in surveyed counties/districts across China in 2014. Figure S2. Coverage, number of participants, and pollutant concentrations in surveyed counties/districts across China in 2016. Figure S3. Coverage, number of participants, and pollutant concentrations in surveyed counties/districts across China in 2018. Table S1. Estimation results of the effects of PM2.5 on the total medical costs. Table S2. Estimation results of the effects of ground surface ozone on the total medical costs. Table S3. Various margin effects for the key independent variables in the model of Tobit-CRE-CF. Table S4. Heterogeneity analysis. [file 12916_2023_2839_MOESM1_ESM.docx]

**Additional file**

**Identifying the causal effects of long-term exposure to PM_2.5_ and ground surface ozone on individual medical costs in China – Evidence from a representative longitudinal nationwide cohort**

Ke Ju, Liyong Lu, Jingguo Yang, Ting Chen, Tianjiao Lan, Zhongxin Duan, Zongyou Xu, En Zhang, Wen Wang, Jay Pan

**Text of contents**

Text S1. Description of covariates

Text S2. Details of Tobit-CRE-CF

**Figure of contents**

Figure S1. Coverage, number of participants, and pollutant concentrations in surveyed counties/districts across China in 2014

Figure S2. Coverage, number of participants, and pollutant concentrations in surveyed counties/districts across China in 2016

Figure S3. Coverage, number of participants, and pollutant concentrations in surveyed counties/districts across China in 2018

**Table of contents**

Table S3. Various margin effects for the key independent variables in the model of Tobit-CRE-CF

Table S1. Estimation results of the effects of PM_2.5_ on the total medical costs

Table S2. Estimation results of the effects of ground surface ozone on the total medical costs

Table S4 Heterogeneity analysis

**Supplementary Text**

**Text S1 Description of covariates**

In this paper, in order to obtain stable results, we control for a range of participant characteristics, specifically including:

(1) *age.* Recorded the participant's age at the time of the interview as a continuous variable with a minimum unit of 1 year.

(2) *gender.* Recorded the gender of the participant at the time of the interview. It is divided into male and female.

(3) *Number of chronic diseases*. The number of chronic diseases that the participant had at the time of the interview was recorded. The categories were Zero, One, Two or more.

(4) *CES-D scores.* The depression scores of participants recorded using the CES-D scale for the year of visit were recorded as a continuous variable, with higher scores representing more severe depression. The reliability of the scale has been previously validated and the Cronbach’s α is 0.86, demonstrating good consistency.

(5) *Whether healthier than last year*. The self-rated comparisons of participants’ physical condition relative to a year ago were recorded and categorized as Healthier, No change and Worse.

(6) *Whether hospitalized last year*. Recorded whether the respondent had been hospitalized due to illness or accidental injury in the year prior to the interview (hospitalization was defined as admission to a hospital room for at least one night)

(7) *Subjective memory impairment*. The self-rated comparisons of participants’ self-rated memory impairment and categorized them as Very well, Well, Not bad, Bad and Very bad based on whether they could remember the main events that occurred during the week.

(8) *Medical insurance*. Recorded the health insurance status of the participant at the time of the interview. According to the China-specific medical insurance system, there are Urban Employment Basic Medical Insurance (UEBMI), Urban Residents Basic Medical Insurance (URBMI), New Cooperative Medical Scheme (NCMS), and Others (i.e., free medical care, another supplement insurance).

(9) *Marriage status*. Recorded the participants’ marriage status and categorized them as Married, Single and Divorce/Widowed.

(10) *Household cooking fuel*. The type of fuel used for cooking in the household was recorded and was categorized as Clean and Unclean according to whether it was clean energy.

(11) *Type of house*. The type of house of participants was recorded and was categorized as One-story and Multi-story house.

(12) *Whether obtaining any subsidy*. Recorded whether the respondent's household received any type of government transfer income, such as minimum subsistence allowance, special hardship allowance, relief, etc., in the year prior to the interview.

(13) *Work status*. The status of work of participants was recorded and was categorized as Unemployed, Employed and Retired.

(14) *Habit of surfing the internet*. Recorded whether the participant reported using any device to access the Internet at the time of the interview.

(15) *Habit of drink*. Respondents' drinking habits were recorded and categorized into those who drank more than or equal to three times per week and those who drank less than three times per week.

(16) *Habit of smoking*. Respondents' smoking habits were recorded and categorized.

(17) *Exercise habits*. Respondents' exercise habits were recorded and categorized according to the times they exercise every week.

(18) *Household per capita income quantile*. The annual per capita household income of the participants at the time of the interview was recorded in the interquartile range of all CFPS respondents in that year. It was categorized into 0-25%, 25-50%, 50-75%, and 75-100% based on income from lowest to highest.

**Text S2 Details of Tobit-CRE-CF.**

Based on the distribution of the response variable in this study, the Tobit regression model is used as the basic model, and the basic Tobit model in this study was designed as follows:

|  | (S1) |
| --- | --- |
|  |  |

where *i* denotes the individual, and *t* the time (Year); *y^*^* is the latent measure of the individual total medical costs; ***X*** is the group of independent variables, including the proxy of air pollutants (PM_2.5_ and ground surface ozone), then key independent variables, and a set of covariates (also called confounders); $\beta$ is the coefficients to be estimated. $\mu$is a random effect term. *y* is the observed individual total medical costs.

The estimation method for the Tobit regression model is the maximum likelihood estimate (MLE). Specifically, the estimation process can be summarized in three steps: (1) Obtain the probability density function for *y* in equation (1); (2) Obtain the likelihood function based on the probability density function. (3) Obtain the parameters using the Newton-Raphson method to maximize the value of the likelihood function.

The probability density function for *y* in equation (S1) in the main text is as follows:

| When *y_it_*=0:   | (S2) |
| --- | --- |
| When *y_it_*>0:   | (S3) |

Based on equations (S2) and (S3), the probability density function for *y* can be formulated in equation (S4).

|  | (S4) |
| --- | --- |

Where *I* represent the indicator function. When the condition is true, the indicator function equals 1, otherwise 0.

The likelihood function is as follows:

|  | (S5) |
| --- | --- |

For formula (S5), the Newton-Raphson method is often used to estimate parameters to make the likelihood function (*logL*) is maximized.

The Tobit regression model is a useful benchmark, yet can be biased by time-invariant and individual-specific effects, and endogeneity.

As a second benchmark, correlated random effects (CRE) were employed to avoid the estimated bias caused by time-invariant and individual-specific effects. This empirical strategy could adjust for unobserved time-invariant and individual-specific effects potentially correlated with both air pollution exposure and the respondents’ health conditions. The key assumption of the CRE regression model is that the unobserved time-invariant and individual-specific effects can be denoted by the combination of independent variables. The unobserved time-invariant and individual-specific effects can be avoided by controlling the combination of independent variables in the regression model. Especially, the model is as follows:

| $y_{it}^{*}=\gamma_{0}+{\boldsymbol{X}_{\boldsymbol{it}}}^{\boldsymbol{'}}\boldsymbol{\theta+}v_{i}+\mu_{it}$  $v_{i}=\alpha+{{\bar{\boldsymbol{X}}}_{i}}^{'}\boldsymbol{\rho}+\varepsilon_{i}$  ${Cov({{\bar{\boldsymbol{X}}}_{i}}^{\boldsymbol{T}},\varepsilon}_{i})=0$ | (S6) |
| --- | --- |

Where $y_{it}^{*}$ is the latent measure of the respondent’s total medical costs, and its detailed explanation sees formula (1). $v_{i}$ is the observed time-invariant and individual-specific effects.${\bar{\boldsymbol{X}}}_{i}$ are the mean values of $\boldsymbol{X}_{\boldsymbol{it}}$ at different years. The basic model is the Tobit regression model, and the specific explanation, including the estimate methods can be seen in formula (S1)-(S5).

We can simplify equation (S6) furtherly:

| $y_{it}=\gamma_{0}+{\boldsymbol{X}_{\boldsymbol{it}}}^{\boldsymbol{'}}\boldsymbol{\theta+}\alpha+{{\bar{\boldsymbol{X}}}_{i}}^{'}\rho+\varepsilon_{i}+\mu_{it}$ | (S7) |
| --- | --- |

Since both$\varepsilon_{i}$ and $\mu_{it}$ are independent of the control variables in formula (7), the composite error term, namely $(\vartheta_{i}+\varepsilon_{it})$, is also uncorrelated with various control variables. The biases caused by unobserved time-invariant and individual-specific effects are avoided by CRE.

The Tobit regression model combined with CRE can avoid the bias caused by unobserved time-invariant and individual-specific effects, while the endogeneity bias potentially caused by a bi-directional association between air pollution exposure and the subject’s health conditions or omitted variables still exists. Previous studies have shown that local air pollutant levels have the potential to influence the subsequent migratory behavior of residents, which is known as “Chasing Clean Air”. For example, high-level air pollutants exposure would damage individual’s health status, and people suffering from diseases would choose to migrate from cities with serious air pollution to cities with better air quality weather conditions.

The instrumental variable method was a good empirical strategy to avoid the bias caused by potential endogeneity, and it is often estimated by the two-stage least squares method. For the Tobit regression model, however, two-stage least-squares estimation is not applicable. In this case, the control function method (also called two-stage residual inclusion) is the best choice. The control function (CF) can correct endogeneity problems by modeling the endogeneity in the residual. The Tobit regression model combined with CRE and CF would be robust to the bias caused by unobserved time-invariant and individual-specific effects in panel data and potentially endogeneity.

Specifically, the process can be divided into three steps: (1) The first step is a similar OLS-CRE of endogenous variable (***Air pollution***) explained by instrumental variables (***Z***), a set of covariates (***X***). To control the unobserved time-invariant and individual-specific fixed effects, based on the principle of CRE described above, a linear combination of instrumental variables (***Z***), covariates (***X***), and endogenous variables were also controlled in the first step. The model of the first step is shown in the formula (S8). (2) Since the CF can avoid the endogeneity bias by modeling the endogeneity in the residual, the second step is to fit the residuals of endogenous variables based on the model in the first step. (3) The third step is to fit the left-censored Tobit regression model combine with CF and CRE for the dependent variable (the total medical costs), corner solution response, explained by the fitted residuals of endogenous variables from the model in the first step (*V*), endogenous variables, a set of covariables (***X***), as well as time-invariant and individual-specific fixed effects explained by a combination of endogenous variable, instrumental variables (***Z***) and included instruments (***X***). The model of the third step is displayed in equation (S9).

| ${Air pollutants}_{it}=g\left( Z,X \right)+\eta_{1}\bar{Air pollutants}_{i}+V$  $where:g\left( Z,X \right)=\beta_{0}+{\boldsymbol{Z}^{\boldsymbol{T}}}_{\boldsymbol{it}}\boldsymbol{\alpha+}{\boldsymbol{X}_{\boldsymbol{it}}}^{\boldsymbol{T}}\boldsymbol{\theta}+\boldsymbol{+}{{\bar{\boldsymbol{Z}}}_{\boldsymbol{i}}}^{\boldsymbol{T}}\boldsymbol{\tau}+{{\bar{\boldsymbol{X}}}_{i}}^{\boldsymbol{T}}\boldsymbol{\omega}$ | (S8) |
| --- | --- |
| ${y^{*}}_{it}=\gamma_{0}+\gamma_{1}{Air pollutants}_{it}+\xi V\boldsymbol{+X}^{\boldsymbol{T}}\boldsymbol{\theta}{\boldsymbol{+}\mu_{i}+\epsilon}_{it}$  $where:\mu_{i}=\alpha+\varphi_{1}\bar{Air pollutants}_{i}+{{\bar{\boldsymbol{Z}}}_{\boldsymbol{it}}}^{\boldsymbol{T}}\boldsymbol{+}{{\bar{\boldsymbol{X}}}_{i}}^{\boldsymbol{T}}\boldsymbol{\rho}+\vartheta_{i}$ | (S9) |

Where ***Air pollutants*** denote the PM_2.5_ and ground surface ozone, ***Z*** represents the instrumental variables, ***X*** is a set of covariates; ***V*** is the residuals of endogenous variables fitted by the model (6); *y^*^* is the latent measure of the respondent’s total medical costs, and its detailed explanation sees formula (S1); $\mu_{i}$ is the unobserved time-invariant and individual-specific fixed effects, it can be explained by a linear combination of endogeneity variables ($\bar{Air pollutants}_{i}$), instrumental variables (${{\bar{\boldsymbol{Z}}}_{\boldsymbol{i}}}^{\boldsymbol{T}}$), and a set of covariates (${{\bar{\boldsymbol{X}}}_{i}}^{\boldsymbol{T}})$; $\epsilon_{it}$ and $\vartheta_{i}$ are the random disturb items.

The estimations of CRE-CF are robust to endogeneity or unbiased only if the instrumental variables (***Z***) can adequately explain variations in air pollution exposure (relevant prerequisite) and also lack the ability to independently explain variations in depression (valid prerequisite). The Cragg-Donald Wald F test was employed to verify whether the instrumental variables (Z) are strongly relevant to the endogenous variables (PM_2.5_ and ground surface ozone) (relevant prerequisite), and the Cragg-Donald Wald F statistic is reported in this study. The Stock-Yogo weak ID test critical value of 10% is used as the criterion, and the relevant prerequisite is valid when the statistic is greater than it. Since the Cragg-Donald Wald F statistic cannot be calculated in the Tobit regression model, we directly fitted the linear regression model for the response variables, namely the respondent’s total medical costs, instead of the Tobit regression model. The valid prerequisite, however, cannot be tested directly from empirical strategy or statistical methods. In previous studies, the Sargan test was used to verify the overidentification or valid prerequisite with the null hypothesis of no overidentification indirectly. The statistics of the Sargan test obey the Chi-squared distribution.

To test or determine whether unobserved time-invariant and individual-specific effects and potential endogeneity exit, we employed the Hausman specification tests in this study with the null hypothesis that the differences in the estimates are not systematic. Specifically, we compared the difference between the Tobit-CRE estimations and the Tobit regression estimation by the Hausman specification tests. If the null hypothesis is rejected, it is suggested that the estimates of Tobit regression estimation would be biased by the unobserved time-invariant and individual-specific effects. In this case, the estimations of Tobit regression would be biased, while the CRE-CF consistent. The estimation of the residuals of PM_2.5_ in equation (9) can be used to test whether the endogeneity exists. If the estimation is statistically significant, the endogeneity of PM_2.5_ exist and would cause bias. In this case, the estimation of Tobit-CRE would be inconsistent, and Tobit-CRE-CF would be consistent.

**Supplementary Figures**

**
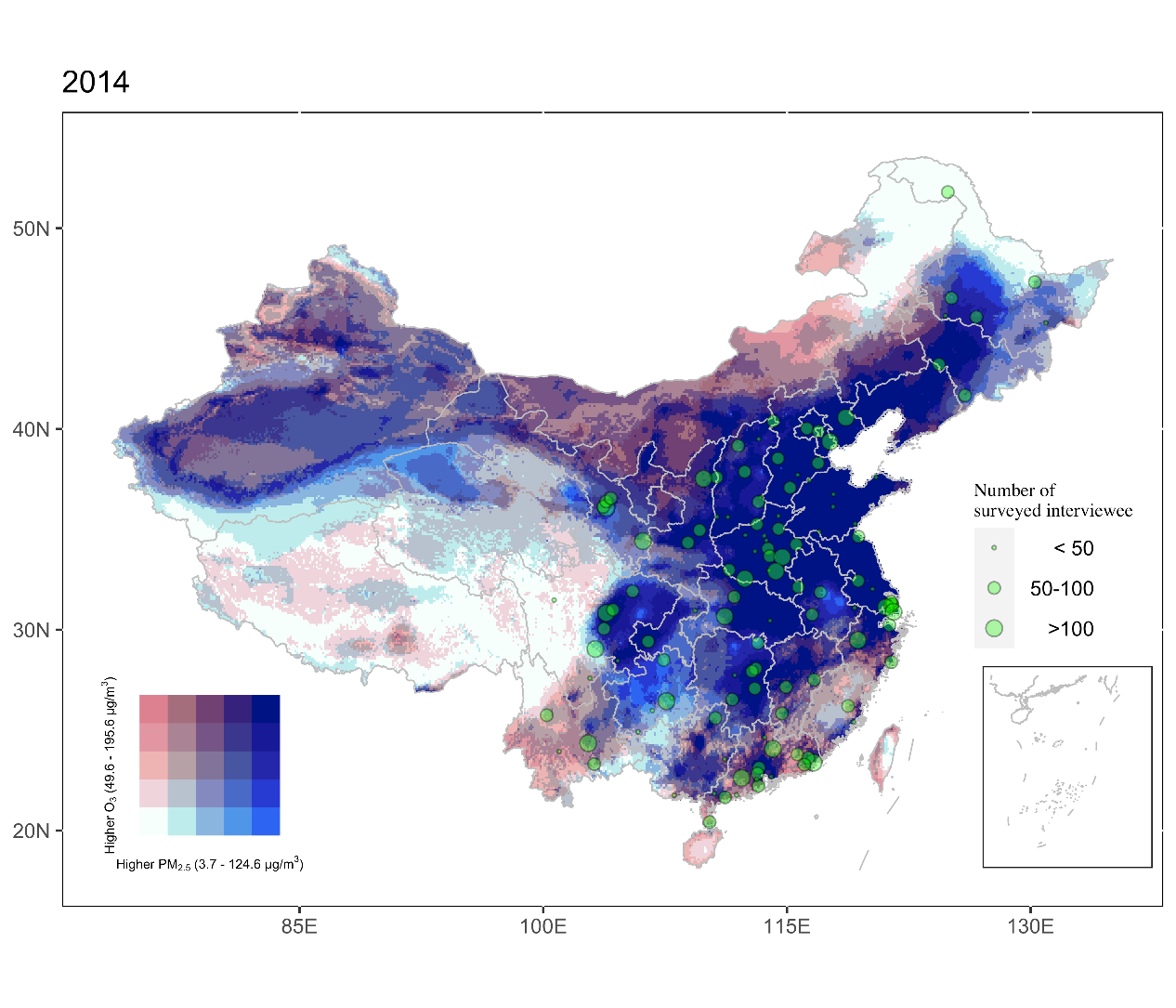
**

**Figure S1.** Coverage, number of participants, and pollutant concentrations in surveyed counties/districts across China in 2014


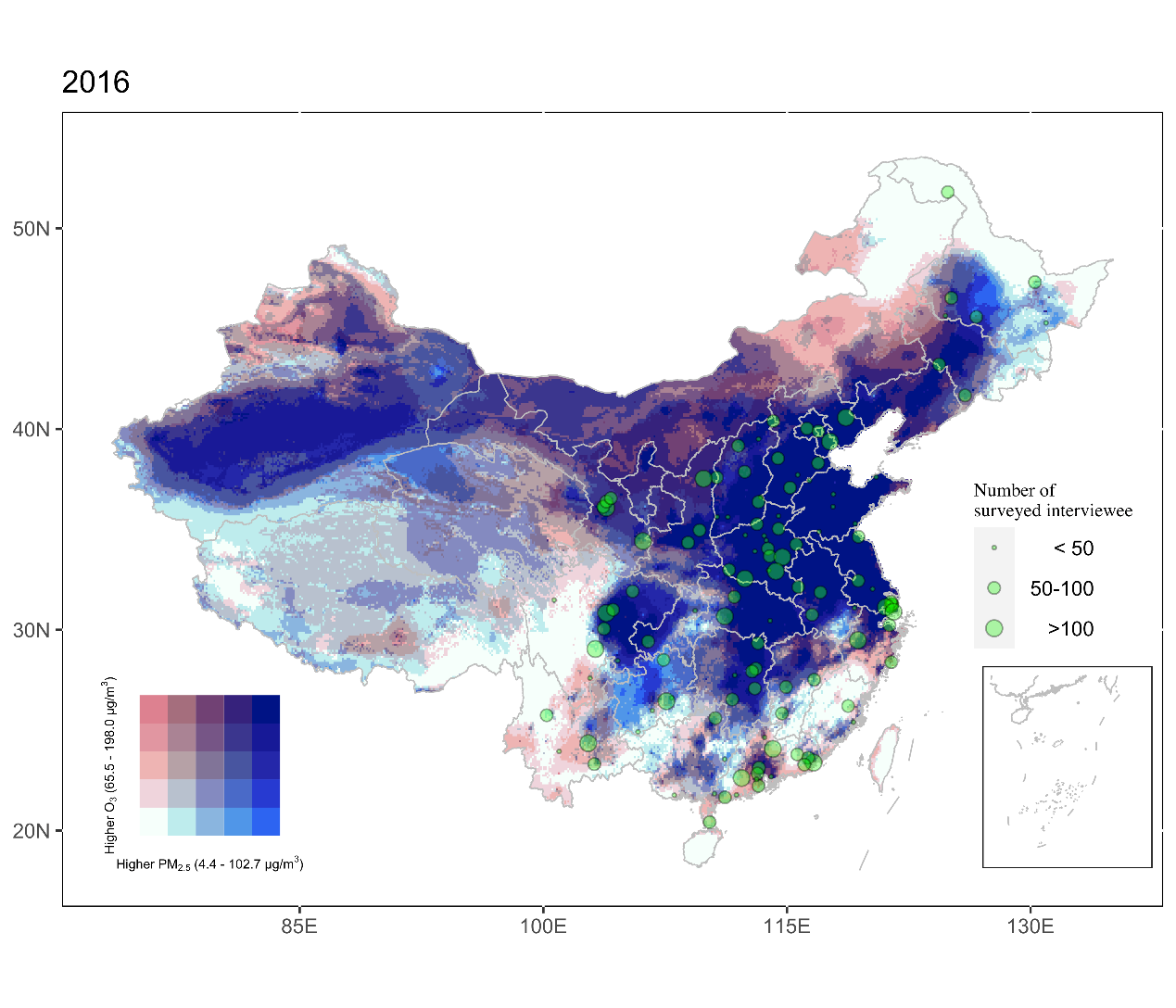


**Figure S2.** Coverage, number of participants, and pollutant concentrations in surveyed counties/districts across China in 2016


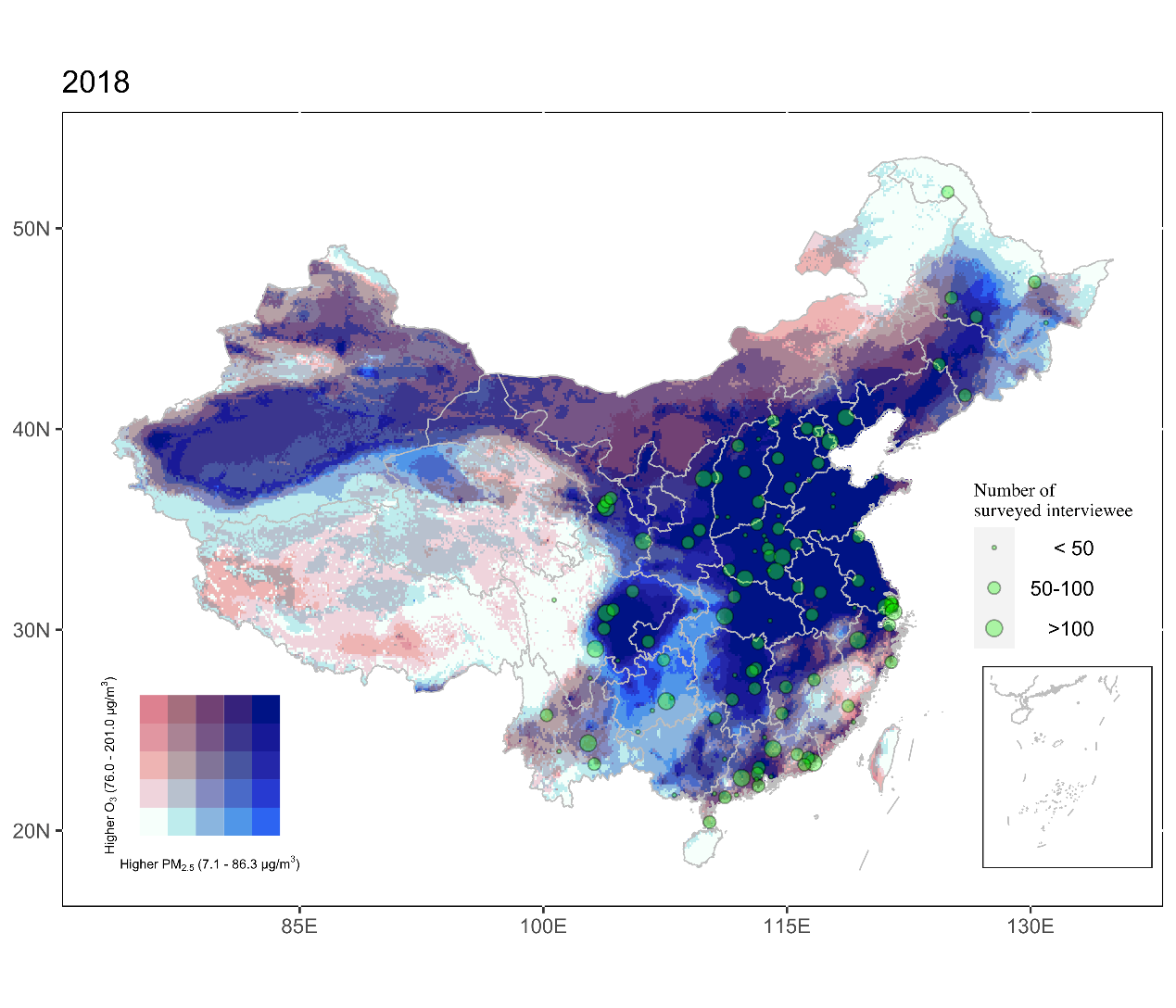


**Figure S3.** Coverage, number of participants, and pollutant concentrations in

surveyed counties/districts across China in 2018

**Supplementary Tables**

**Table S1** Estimation results of the effects of PM_2.5_ on the total medical costs

| Variables | ln (Total medical costs) | | Total medical costs | | |
| --- | --- | --- | --- | --- | --- |
|  | (1) | (2) | (3) | (4) | (5) |
|  | Linear regression | Fixed-effects 2SLS | Pool-Tobit | Tobit-CRE | Tobit-CRE-CF |
| PM_2.5_ | -0.008*** | 0.052 | -16.527*** | -47.180* | 526.396* |
|  | (0.001) | (0.048) | (4.598) | (18.675) | (232.711) |
| PM_2.5__residual | - | - | - | - | -576.490* |
|  | - | - | - | - | (230.521) |
| Age | 0.020*** | 0.140 | 35.971*** | 597.993 | 573.123 |
|  | (0.002) | (0.093) | (8.432) | (494.746) | (482.278) |
| Gender (Ref=female) |  |  |  |  |  |
| Male | -0.377*** | - | 169.350 | 81.197 | 93.306 |
|  | (0.045) | - | (210.474) | (221.907) | (263.964) |
| Number of chronic diseases (Ref=zero) |  |  |  |  |  |
| One | 1.726*** | 1.132*** | 3,644.217*** | 2,750.239*** | 2,763.355*** |
|  | (0.054) | (0.063) | (242.561) | (274.685) | (430.262) |
| Two or more | 1.713*** | 0.951*** | 3,403.238*** | 1,665.924*** | 1,732.185*** |
|  | (0.064) | (0.078) | (287.485) | (384.982) | (421.266) |
| CESD scores (Depression symptom) | 0.039*** | 0.009 | 115.685*** | 74.962*** | 31.848 |
|  | (0.003) | (0.005) | (12.304) | (17.998) | (25.824) |
| Whether healthier than last year (Ref=healthier) |  |  |  |  |  |
| No change | -0.363*** | -0.130 | -2,456.797*** | -2,018.694*** | -2,067.382*** |
|  | (0.060) | (0.071) | (285.525) | (308.523) | (452.123) |
| Worse | 0.620*** | 0.563*** | -172.550 | 571.459 | 555.865 |
|  | (0.063) | (0.076) | (296.571) | (385.314) | (536.308) |
| Whether hospitalized last year (Ref=No) |  |  |  |  |  |
| Yes | 3.425*** | 3.140*** | 15,005.371*** | 14,388.826*** | 14,194.258*** |
|  | (0.052) | (0.064) | (234.710) | (314.316) | (607.550) |
| Subjective memory status (Ref=Very bad) |  |  |  |  |  |
| Bad | 0.241*** | 0.126 | 650.404** | 759.261** | 583.653* |
|  | (0.052) | (0.065) | (248.682) | (251.605) | (296.088) |
| Not bad | 0.247*** | 0.191*** | 596.125* | 731.779** | 824.964** |
|  | (0.049) | (0.057) | (235.033) | (245.315) | (270.726) |
| Well | 0.261*** | 0.185** | 767.360** | 953.562*** | 1,237.037*** |
|  | (0.056) | (0.064) | (265.868) | (283.736) | (286.747) |
| Very well | 0.128* | 0.069 | 43.769 | 348.517 | 546.230 |
|  | (0.061) | (0.065) | (291.703) | (324.777) | (348.055) |
| Medical insurance (Ref=UEBMI) |  |  |  |  |  |
| URBMI | -0.332*** | -0.102 | 142.216 | -648.919 | -714.389 |
|  | (0.074) | (0.107) | (350.703) | (397.152) | (608.661) |
| NCMS | -0.209*** | -0.166 | -373.447 | -2,423.569*** | -2,556.600** |
|  | (0.060) | (0.133) | (284.013) | (543.850) | (791.450) |
| Others | -0.071 | -0.140 | 1,138.291* | -824.040 | -1,137.165 |
|  | (0.115) | (0.149) | (545.380) | (711.108) | (1,087.606) |
| Marriage status (Ref=Married) |  |  |  |  |  |
| Single | 0.092 | 0.167 | 567.427 | 1,307.056* | 1,166.741 |
|  | (0.107) | (0.230) | (513.842) | (655.275) | (623.189) |
| Divorce/Widowed | -0.072 | 0.371* | -702.610* | 867.954 | 772.369 |
|  | (0.069) | (0.174) | (322.739) | (885.365) | (772.576) |
| Household cooking fuel (Ref=clean) |  |  |  |  |  |
| Unclean | 0.108** | 0.009 | 93.206 | -192.008 | -222.046 |
|  | (0.042) | (0.064) | (197.027) | (338.399) | (246.225) |
| Type of house (Ref=One-story) |  |  |  |  |  |
| Multi-story | 0.131** | 0.121* | 259.662 | 163.197 | 228.410 |
|  | (0.040) | (0.055) | (189.383) | (245.207) | (220.459) |
| Unknown | 0.073 | -0.017 | 248.674 | 13.335 | -6.758 |
|  | (0.064) | (0.077) | (302.219) | (393.196) | (383.813) |
| Whether obtaining any subsidy (Ref=Yes) |  |  |  |  |  |
| No | 0.310*** | 0.188** | 771.299*** | 526.019 | 238.514 |
|  | (0.039) | (0.058) | (184.220) | (278.461) | (355.358) |
| Work status (Ref=Unemployed) |  |  |  |  |  |
| Employed | 0.467* | 0.119 | 1,151.946 | 1,261.078 | 1,463.419 |
|  | (0.227) | (0.258) | (1,106.291) | (1,105.294) | (935.216) |
| Retired | 0.712** | 0.334 | 3,625.399** | 3,463.940** | 3,994.939*** |
|  | (0.230) | (0.264) | (1,118.562) | (1,158.237) | (1,072.547) |
| Unknown | 0.647 | 0.663 | 2,232.273 | 2,254.916 | 3,438.520* |
|  | (0.439) | (0.580) | (2,098.313) | (2,136.635) | (1,632.410) |
| Habit of surfing the internet (Ref=Yes) |  |  |  |  |  |
| No | 0.091 | -0.034 | 223.561 | -320.207 | -125.860 |
|  | (0.047) | (0.066) | (224.681) | (344.628) | (452.591) |
| Habit of drinking (Ref=No) |  |  |  |  |  |
| Yes | -0.376*** | -0.125 | -1,278.793*** | -657.615 | -581.487 |
|  | (0.050) | (0.078) | (241.470) | (415.696) | (455.801) |
| Whether smoking (Ref=No) |  |  |  |  |  |
| Yes | -0.127** | -0.049 | -841.118*** | -2,641.861*** | -2,313.388*** |
|  | (0.046) | (0.104) | (221.467) | (529.716) | (648.849) |
| Exercise habits (Ref=Frequently) |  |  |  |  |  |
| Occasionally | 0.105 | 0.107 | 22.568 | -1.044 | 168.175 |
|  | (0.056) | (0.068) | (263.714) | (280.041) | (318.524) |
| Hardly/never | -0.047 | -0.005 | -4.602 | -129.717 | 323.456 |
|  | (0.042) | (0.066) | (199.317) | (284.602) | (360.160) |
| Household per capita income quantile (Ref=0-25%,1st) |  |  |  |  |  |
| *25-50%, 2_nd_* | 0.158*** | 0.110 | 610.451** | 544.618* | 653.733** |
|  | (0.048) | (0.059) | (224.964) | (246.614) | (248.625) |
| *50-75%, 3_rd_* | 0.121* | 0.154* | 736.450** | 703.367* | 793.425* |
|  | (0.051) | (0.068) | (241.346) | (322.318) | (320.429) |
| *75-100%, 4_th_* | 0.277*** | 0.241** | 1,736.080*** | 1,614.876*** | 1,724.849** |
|  | (0.061) | (0.082) | (287.935) | (427.912) | (599.614) |
| City per capita income | 0.000*** | -0.000 | 0.041*** | 0.016 | 0.064* |
|  | (0.000) | (0.000) | (0.006) | (0.019) | (0.030) |
| City per capita hospital beds | -0.007*** | 0.006 | -25.780*** | 13.831 | 52.084* |
|  | (0.001) | (0.004) | (5.845) | (16.522) | (22.739) |
| Year dummy | Yes | Yes | Yes | Yes | Yes |
| Correlated random effects | No | No | No | Yes | Yes |
| *N* | 26,784 | 26,784 | 26,784 | 26,784 | 26,784 |
| *R^2^* | 0.342 | 0.180 | - | - | - |
| Cragg-Donald Wald F statistic | - | 48.229>19.93 | - | - | - |
| Sargan statistic | - | 1.245 (*P*=0.265>0.05) | - | - | - |

Note: (1) Standard errors in parentheses; (2) Correlated random effects: a linear combination of endogenous variables, instrumental variables and covariates; (3) * p < 0.05, ** p < 0.01, *** p < 0.001.

**Table S2** Estimation results of the effects of ground surface ozone on the total medical costs

| Variables | ln (total medical fees) | | Total medical fees | | |
| --- | --- | --- | --- | --- | --- |
|  | (1) | (2) | (3) | (4) | (5) |
|  | Linear regression | Fixed-effects 2SLS | Pool-Tobit | Tobit-CRE | Tobit-CRE-CF |
| Ground surface ozone | -0.009*** | 0.023 | -4.859 | 46.420** | 198.626* |
|  | (0.001) | (0.019) | (6.752) | (16.254) | (87.428) |
| Ozone_residual | - | - | - | - | -156.447 |
|  | - | - | - | - | (91.514) |
| Age | 0.020*** | 0.126 | 35.299*** | 558.272 | 456.924 |
|  | (0.002) | (0.094) | (8.444) | (494.611) | (451.457) |
| Gender (Ref=female) |  |  |  |  |  |
| Male | -0.379*** | - | 183.490 | 85.269 | 79.770 |
|  | (0.045) | - | (210.514) | (221.989) | (258.669) |
| Number of chronic diseases (Ref=zero) |  |  |  |  |  |
| One | 1.723*** | 1.133*** | 3,643.355*** | 2,754.952*** | 2,752.433*** |
|  | (0.054) | (0.063) | (242.611) | (274.680) | (425.188) |
| Two or more | 1.704*** | 0.945*** | 3,389.020*** | 1,668.125*** | 1,671.378*** |
|  | (0.064) | (0.077) | (287.500) | (384.959) | (406.568) |
| CESD scores (Depression symptom) | 0.039*** | 0.013*** | 117.799*** | 72.227*** | 74.787*** |
|  | (0.003) | (0.003) | (12.318) | (17.947) | (21.289) |
| Whether healthier than last year (Ref=healthier) |  |  |  |  |  |
| No change | -0.370*** | -0.117 | -2,483.775*** | -2,029.959*** | -2,021.110*** |
|  | (0.060) | (0.070) | (285.458) | (308.438) | (469.584) |
| Worse | 0.621*** | 0.567*** | -167.850 | 562.274 | 558.331 |
|  | (0.063) | (0.075) | (296.585) | (385.276) | (544.333) |
| Whether hospitalized last year (Ref=No) |  |  |  |  |  |
| Yes | 3.412*** | 3.167*** | 14,986.321*** | 14,388.240*** | 14,443.808*** |
|  | (0.052) | (0.062) | (234.723) | (314.300) | (599.093) |
| Subjective memory status (Ref=Very bad) |  |  |  |  |  |
| Bad | 0.238*** | 0.175** | 660.815** | 773.496** | 841.087** |
|  | (0.052) | (0.060) | (248.754) | (251.582) | (277.740) |
| Not bad | 0.251*** | 0.189*** | 611.476** | 742.863** | 730.985** |
|  | (0.049) | (0.056) | (235.014) | (245.272) | (271.826) |
| Well | 0.267*** | 0.157** | 780.780** | 961.451*** | 921.884** |
|  | (0.056) | (0.060) | (265.856) | (283.669) | (292.557) |
| Very well | 0.132* | 0.055 | 56.561 | 355.260 | 319.478 |
|  | (0.062) | (0.064) | (291.699) | (324.741) | (341.650) |
| Medical insurance (Ref=UEBMI) |  |  |  |  |  |
| URBMI | -0.323*** | -0.103 | 186.183 | -629.976 | -641.729 |
|  | (0.074) | (0.106) | (350.562) | (397.064) | (601.873) |
| NCMS | -0.217*** | -0.162 | -364.421 | -2,434.803*** | -2,386.516** |
|  | (0.060) | (0.132) | (284.249) | (543.909) | (789.329) |
| Others | -0.059 | -0.092 | 1,171.717* | -817.926 | -758.397 |
|  | (0.115) | (0.145) | (545.324) | (711.090) | (1,116.875) |
| Marriage status (Ref=Married) |  |  |  |  |  |
| Single | 0.112 | 0.232 | 614.694 | 1,336.082* | 1,404.623* |
|  | (0.107) | (0.225) | (513.670) | (655.190) | (601.007) |
| Divorce/Widowed | -0.075 | 0.378* | -685.607* | 869.088 | 930.833 |
|  | (0.069) | (0.173) | (322.786) | (885.305) | (721.274) |
| Household cooking fuel (Ref=clean) |  |  |  |  |  |
| Unclean | 0.132** | 0.007 | 204.795 | -207.745 | -241.698 |
|  | (0.041) | (0.064) | (195.786) | (338.415) | (232.656) |
| Type of house (Ref=One-story) |  |  |  |  |  |
| Multi-story | 0.122** | 0.123* | 282.473 | 179.802 | 257.051 |
|  | (0.040) | (0.055) | (190.169) | (245.356) | (217.748) |
| Unknown | 0.062 | -0.001 | 261.794 | 43.569 | 112.133 |
|  | (0.064) | (0.077) | (302.648) | (393.366) | (391.005) |
| Whether obtaining any subsidy (Ref=Yes) |  |  |  |  |  |
| No | 0.293*** | 0.219*** | 740.139*** | 512.623 | 539.149 |
|  | (0.039) | (0.052) | (184.076) | (278.300) | (314.873) |
| Work status (Ref=Unemployed) |  |  |  |  |  |
| Employed | 0.481* | 0.100 | 1,182.306 | 1,289.738 | 1,271.235 |
|  | (0.227) | (0.255) | (1,106.559) | (1,105.343) | (916.116) |
| Retired | 0.736** | 0.281 | 3,662.705** | 3,505.241** | 3,441.783*** |
|  | (0.230) | (0.258) | (1,118.854) | (1,158.161) | (1,037.727) |
| Unknown | 0.693 | 0.411 | 2,303.536 | 2,250.804 | 1,900.152 |
|  | (0.439) | (0.566) | (2,098.285) | (2,136.727) | (1,505.092) |
| Habit of surfing the internet (Ref=Yes) |  |  |  |  |  |
| No | 0.094* | -0.059 | 246.649 | -321.138 | -374.470 |
|  | (0.047) | (0.064) | (224.672) | (344.622) | (428.906) |
| Habit of drinking (Ref=No) |  |  |  |  |  |
| Yes | -0.372*** | -0.134 | -1,284.719*** | -658.388 | -668.789 |
|  | (0.050) | (0.077) | (241.535) | (415.685) | (451.319) |
| Whether smoking (Ref=No) |  |  |  |  |  |
| Yes | -0.119* | -0.088 | -821.295*** | -2,637.189*** | -2,697.440*** |
|  | (0.046) | (0.099) | (221.422) | (529.594) | (608.513) |
| Exercise habits (Ref=Frequently) |  |  |  |  |  |
| Occasionally | 0.110* | 0.092 | 22.625 | 6.916 | -26.933 |
|  | (0.056) | (0.067) | (263.748) | (280.009) | (326.970) |
| Hardly/never | -0.042 | -0.056 | -0.887 | -111.668 | -176.751 |
|  | (0.042) | (0.054) | (199.341) | (284.297) | (319.305) |
| Household per capita income quantile (Ref=0-25%,1st) |  |  |  |  |  |
| *25-50%, 2_nd_* | 0.163*** | 0.095 | 606.733** | 547.830* | 570.117* |
|  | (0.048) | (0.057) | (225.040) | (246.566) | (246.264) |
| *50-75%, 3_rd_* | 0.140** | 0.146* | 761.516** | 721.092* | 766.123* |
|  | (0.051) | (0.067) | (241.374) | (322.315) | (312.638) |
| *75-100%, 4_th_* | 0.303*** | 0.245** | 1,767.980*** | 1,654.462*** | 1,739.435** |
|  | (0.061) | (0.082) | (288.059) | (427.985) | (599.518) |
| City per capita income | 0.000*** | -0.000 | 0.044*** | 0.013 | -0.008 |
|  | (0.000) | (0.000) | (0.006) | (0.020) | (0.024) |
| City per capita hospital beds | -0.007*** | 0.002 | -27.959*** | 14.740 | 7.189 |
|  | (0.001) | (0.003) | (5.850) | (16.499) | (14.595) |
| Year dummy | Yes | Yes | Yes | Yes | Yes |
| Correlated random effects | No | No | No | Yes | Yes |
| *N* | 26,784 | 26,784 | 26,784 | 26,784 | 26,784 |
| *R^2^* | 0.341 | 0.192 | - | - | - |
| Cragg-Donald Wald F statistic | - | 241.401>19.93 | - | - | - |
| Sargan statistic | - | 0.898(*P*=0.343>0.05) | - | - | - |

Note: (1) Standard errors in parentheses; (2) Correlated random effects: a linear combination of endogenous variables, instrumental variables and covariates; (3) * p < 0.05, ** p < 0.01, *** p < 0.001.

**Table S3** Various margin effects for the key independent variables in the model of Tobit-CRE-CF

| Variables | Margin effects |  |  |
| --- | --- | --- | --- |
|  | *y^*^* | *y^*^\|y>0* | *y\|y>0* |
| PM_2.5_ | 526.396* | 256.532* | 199.144* |
|  | (254.202) | (123.886) | (96.174) |
| Ground surface ozone | 198.626* | 96.803* | 75.145* |
|  | (99.114) | (48.310) | (37.499) |

Note: (1) Standard errors in parentheses; (2) * p < 0.05, ** p < 0.01, *** p < 0.001.

**Table S4** Heterogeneity analysis

| Variable | (1) | (16) | (17) | (18) |
| --- | --- | --- | --- | --- |
|  | Gender | | Age | |
|  | PM_2.5_ | Ozone | PM_2.5_ | Ozone |
| PM_2.5_ | 513.722* | - | 526.665* | - |
|  | (232.779) | - | (232.624) | - |
| Ground surface ozone | - | 188.586* | - | 194.985* |
|  | - | (88.156) | - | (87.618) |
| Gender * PM_2.5_ (Ref=Male*PM_2.5_) |  |  |  |  |
| Female*PM_2.5_ | 24.202** | - |  |  |
|  | (7.922) | - |  |  |
| Gender* Ground surface ozone (Ref=Male*PM_2.5_) |  |  |  |  |
| Female* Ground surface ozone | - | 19.885 | - | - |
|  | - | (13.817) | - | - |
| Age*PM_2.5_ | - | - | 0.021 | - |
|  | - | - | (0.280) | - |
| Age* Ground surface ozone |  |  | - | 2.154*** |
|  |  |  | - | (0.531) |
| Other control variables | Yes | Yes | Yes | Yes |
| *N* | 26,784 | 26,784 | 26,784 | 26,784 |

Note: (1) Standard errors in parentheses; (2) Other control variables include a set of covariates and correlated random effects; (3) Interaction items are centralized. (4) * p < 0.05, ** p < 0.01, *** p < 0.001.
